# Transformer-Based Deep Learning for Population-Scale Retinal Image Screening of Ophthalmic Disorders

**DOI:** 10.3390/bioengineering13040377

**Published:** 2026-03-25

**Authors:** Wiem Abdelbaki, Wided Bouchelligua, Inzamam Mashood Nasir, Sara Tehsin, Hend Alshaya

**Affiliations:** 1 College of Engineering and Technology, American University of the Middle East, Egaila 54200, Kuwait; wiem.abdelbaki@aum.edu.kw; 2 Applied College, Imam Mohammad Ibn Saud Islamic University (IMSIU), Riyadh 11432, Saudi Arabia; wabouchelligua@imamu.edu.sa; 3 Human-Environment-Technology (HET) Systems Centre, Mykolas Romeris University, 08303 Vilnius, Lithuania; inzamam.nasir@mruni.eu; 4 Faculty of Informatics, Kaunas University of Technology, 51368 Kaunas, Lithuania

**Keywords:** retinal image screening, diabetic retinopathy, multi-disease classification, vision transformers, population-scale healthcare, medical image analysis

## Abstract

To perform screening of the retina on a population scale, an automated procedure is required that incorporates accurate, reproducible, interpretable, and computationally costeffective models. Existing approaches using convolutional or transformer architectures typically do not adequately represent both fine-grained pathology and large-scale retinal context simultaneously, which could adversely affect their reliability if used for large-scale applications in clinical practice. In this paper, we propose a hierarchical transformer-based screening framework for retinal fundus images that incorporates patch-based tokenization, global transformer encoding, and hierarchical aggregation of contextual information. We also developed a lightweight prediction head that supports screening for both single and multiple diseases. The framework has been evaluated using standard screening metrics, robustness, and cross-dataset generalization analyses on two eye retinopathy image databases: EyePACS and RFMiD. With regard to screening for a binary outcome of diabetic retinopathy, our method provided an accuracy of 89.4% and an area under the receiver operating characteristic (AUROC) curve of 93.6% on EyePACS and attained an accuracy of 95.2% and a macro-averaged F1 score of 82.7% on RFMiD. Our hierarchical transformer achieved improved robustness to degraded images and increased generalizability across datasets compared with all current state-of-the-art models. The proposed hierarchical transformer demonstrates strong potential for large-scale retinal screening and provides a promising foundation for future clinically validated deployment.

## 1. Introduction

Vision-threatening eye diseases such as glaucoma, diabetic retinopathy, and age-related macular degeneration pose a significant global health challenge. They also rank among the leading causes of irreversible blindness worldwide. Large retinal screening programs provide considerable benefits to patients through early diagnosis and timely treatment. However, because assessment of fundus photographs is resource-intensive, as well as there being substantial variability between observers, the ability to scale operations for population-based initiatives is restricted [1].

Digital retinal imaging is growing fast and is thus driving the use of automatic/automated analysis tools that can assist in the screening workflow process. Deep learning has emerged as a robust approach for obtaining clinically meaningful quantitative information from retinal fundus images and to automatically detect ocular diseases and determine the severity level at scale [2]. Automated analysis systems provide great benefits to areas of the world lacking ophthalmologists and where additional demand exists for screening [3].

Automated screening systems for the retina have typically relied upon the use of convolutional neural networks (CNNs) as the main method for performing these kinds of applications. A number of the earlier studies relying upon CNNs were very successful in detecting diabetic retinopathy, thus demonstrating that algorithm-assisted screening was possible with an accuracy rate similar to human grades [4]. Other studies have looked to further enhance the robustness and generalizability aspects of these CNN-based techniques through the use of deeper architectures and better representation of features to help overcome some of the imaging conditions that do not possess adequate representation across the variety of real-world datasets used in retinopathy screening [5,6].

Even though CNNs have an advantage, locally receptive fields are restricting their ability to learn from the entire area of the retina, which may impact how pathology is detected when testing, as pathology can be far apart and also heavily reliant on context, not just locally [7]. To address this issue, attempts at using attention mechanisms and multi-scale aggregation techniques have produced small improvements but have added several design complexities and have not completely solved the global context modeling problems [8].

Due to the self-attention mechanism commonly associated with transformers, they have become very popular in the field of computer vision because they are able to use global attention to model global relationships within an image. Vision transformers use patch-level tokens in the decomposition of an image, as well as apply global attention, thereby enabling direct global long-range spatial interaction to be modeled that cannot be effectively captured using just convolutional neural networks [9]. Overall, these properties make using transformers increasingly attractive for medical imaging tasks that require a holistic understanding of images [10].

Transformers have shown great success in the area of retinal image analysis for solving classification and multi-label detection problems. Transformer’s hierarchical architecture has improved computational efficiency through better use of localized attention in conjunction with increasing receptive field sizes, making these architectures very well suited for use with high-resolution fundus images [11]. Additionally, hierarchies will serve as an advantage for large-scale screening because they provide both scalability and accuracy, which are key aspects of successful population screening [12].

This study introduces a transformer-based hierarchical framework for screening retinal images from a population-wide perspective. This framework identifies pathological cues and the global context of the retina for a single disease or multiple diseases and is computationally efficient for both forms of screening. This scalable framework was tested on two large-scale datasets (EyePACS and RFMiD) to demonstrate its ability to improve the performance of the population health screening process, including improvements to accuracy, macro-F1 score, robustness to degraded images, and the ability to generalize across multiple datasets compared to state-of-the-art CNNs and transformers, suggesting that this framework can be deployed at scale in everyday clinical practice.

The rest of this paper consists of four sections, with Section 2 reviewing related work on the CNN- and transformer-based methods used by current researchers when screening retinal. Section 3 details our proposed hierarchical transformer framework, and the different components. Section 4 presents the results, comparisons to baseline methods, and ablation study conducted during the course of our study. Section 5 provides the conclusions of this research and includes a discussion related to possible future directions for research.

## 2. Related Work

The enormous number of people who suffer from preventable vision loss is making large-scale screening with retinal images into one of the main uses for deep learning in ophthalmology. Additionally, advances in large dataset availability for fundus imaging will contribute consistently to this area as well. Thus far, almost all automated screening systems have been built using CNNs, which have been shown to perform very well with respect to identifying diabetic retinopathy and other conditions related to it from color fundus photos. The ability of this type of approach to provide routine assistance for automated diagnosis in an actual clinical environment has been verified by their ability to show performance similar to that of expert ophthalmologists, despite the fact that these systems were evaluated under controlled conditions [13,14].

Further research extended convolutional neural network (CNN) functionality beyond simple classification of images to increase reliability and generalization among many differences between picture-taking conditions used in population-based screening programs. Specific architectures that have been greatly used after deepening have included the use of dense connections and multi-length feature extraction in an inception-like fashion. All of these were particularly helpful in being able to detect very subtle diseases like microaneurysms, macular hemorrhages and exudates [15,16,17]. The results produced by CNNs on large datasets such as EyePACs have shown an increase in both classification accuracy and area under the receiver operating characteristic curve for the family of CNNs, including ResNet, Inception, and EfficientNet, thereby providing strong performance as baseline models for detecting diabetic retinopathy [18,19]

Notwithstanding these developments, however, the limitations of CNN-based systems continue to persist due to the very nature of local receptive fields, which restricts the capability of CNNs to effectively model global spatial relationship within an image. This is especially important for multi-disease screening, as many of the cues that indicate disease may be distributed and/or contextual. To meet this challenge, we developed hybrid CNN systems that utilize attention mechanisms and feature recalibration strategies, providing an increased sensitivity to areas important for diagnosis without compromising computational efficiency [20,21].

Recently, there has been a new type of architecture called transformers that can replace other forms of architecture for retinal image analysis. Vision transformers (ViTs) use a method of global self-attention to capture long-distance relationships between data and have been trained to be able to handle large amounts of data. Transformer-based models are more durable than traditional pixel-based methods when used in a screening process where thousands of images are taken under varying lighting conditions; they can still show robust performance when tested against degraded images and images taken with different devices [9,22]. Baseline ViT models have consistently outperformed traditional CNN models on large-scale datasets such as Eyepacs, making them ideal for potential deployment worldwide [23,24].

Recent work has moved beyond the basic vision transformer baseline to create more advanced hierarchical and multi-scale structures for retinal image analysis, including window-based transformers, hybrid CNN–transformer designs and efficiency-orientated vision backbones [25,26]. These models help to provide a better balance between the global context that is being modeled and the fine detail required for analyzing lesions, and deliver an efficient way to screen a very large number of patients. The use of a hierarchical aggregation method is largely in line with this area of study and will be focused on increasing the size of the attention context over time and the merging of features from multiple depths of the network, providing better solutions for applications specific to retinal screening [27,28]. A recent trend in the development of technology has been the focus on developing clinically valid and domain-specific AI solutions within healthcare workflows. Several researchers, including [29,30], have developed PatientEase, an augmentation retrieval generation (ARG) framework that is designed to improve the quality of rehabilitation instructions while retaining clinical significance by using a multiagent and dual retrieval model as an example of the growing trend toward the creation of reliable and adaptive medical AI systems. While PatientEase focused on the use of natural language processing to support healthcare providers with clinical documentation versus the analysis of retinal images, it does indicate that there is an increase in the development of clinically valid vision-based screening systems by promoting further clinical development of clinically valid image-processing models.

The evolution of large-scale foundation models has started a transformation in both retinal and more general medical image analysis. For example, there are now large pretrained retinal foundation models that exhibit impressive cross-disease generalization ability as well as robustness to heterogeneous acquisition modalities [31,32]. There are also large generalist medical foundation models that are able to perform open-world segmentation and highlight the scalability of representation learning across the different anatomical parts and modalities involved in the body and its health [33]. Further, in the specific area of hierarchical dense predictions and pyramidal feature learning, which consider multi-scale pathology, these works advance both the hierarchical view of the pathological process and multi-scale representation of pathology in order to present many different aspects of this type of pathology successfully [34]. Weakly supervised and label-efficient architectures offer significant potential for clinical application in settings that have limited access to expert annotators [35,36].

In addition, multimodal integration techniques that use imaging, genetics, histology, and longitudinal clinical data along with cross-modal attention mechanisms have shown promise in prognostic or risk stratification settings [37]. These types of new technologies also reflect a larger trend towards developing one type of AI system as an all-purpose, multimodal, transferable system in the healthcare space. The current study is limited to investigating only structured population-scale retinal screening performed under controlled conditions; however, these emerging paradigms will help to provide additional context to better position the proposed hierarchical transformer within the currently evolving research landscape. Additionally, recent domain-aware retrieval-augmented generation (RAG) strategies have demonstrated promise in tailoring complex clinical instructions and knowledge synthesis across heterogeneous biomedical domains [29], reflecting the growing integration of large-scale language models with domain-specific adaptation techniques in medical AI.

Recent progressions in consistency learning and representation refinement that are data-efficient have given rise to improved robustness in many common medical imaging use cases. Copy-paste consistency methodologies have been investigated again with the aim of enhancing the consistency of segmentation when limited supervision is available [38], showing the importance of the learner’s awareness to augmentation. Generative transformation-based systems are also showing the potential for cross-modal image translations (inter-domain images), as illustrated through the use of phase-contrast and optical coherence tomography images for the purpose of enabling cross-modality adaptation in retinal screening [39]. Additionally, newer diffusion-based architectures and large language model integrated architectures are demonstrating the ability to generalize both reconstruction and representation learning beyond task-specific domains, indicating a move to larger and more transferable AI systems [40].

In addition to single-disease detection, transformer-based architectures have been utilized for performing multi-label retinal screening with several datasets—including the RFMiD that cover various ophthalmic diseases. Transformer models, including the Swin transformer types, have been evaluated against the most advanced CNN architectures, demonstrating comparable performance and better interpretability through attention maps [41,42,43]. This feature makes transformers a strong option for large-scale screening systems needing both high diagnostic performance and clinical transparency. To provide an overview of the accuracy of the current methods, Table 1 below presents a summary of CNN and transformer baseline models currently in use to evaluate the large-scale performance using EyePACS and RFMiD datasets from the most recent research literature. The performance values reported in Table 1 are representative results summarized from the respective original publications and are provided for contextual comparison with prior work. Direct numerical comparison should be interpreted cautiously due to differences in experimental protocols, data preprocessing, and evaluation settings across studies.

CNNs and transformers are making important strides in the field of automated retina image screening. However, there still remain significant limitations to using these types of models for large-scale screening in the general population. CNN-based models rely on local receptive fields that do not provide sufficient long-range anatomical dependencies or allow for pathologies to be widespread throughout the retina when screening for multiple diseases simultaneously. Recent vision transformer models have begun addressing some of the limitations associated with global context modeling; however, they also employ an approach by treating spatial tokens as if they were all identical. As a result, the sensitivity of these models to detecting fine-grained lesions is reduced, there is an increase in computational costs, and the robustness of these models is diminished when there is variability in the quality of the input images or domain shifts between datasets. Finally, virtually all existing methods that are available today only seek to optimize models that can be used for only one disease. Consequently, no existing methods provide sufficient levels of consistency in terms of performance and interpretability for both single-disease and multi-disease screening.

This framework will provide detailed levels of sensitivity in relation to local lesions and help to contextualize the various global retinal structures in terms of how they work as a whole. The overall structure of the framework can help to accumulate contextual information through the transformer blocks so that the transformer networks can cover up the locality deficiencies found in CNN topology and also address some of the inefficiencies from using a flat global attention block within this type of structure. Together with having a unified predictive head that is specifically developed for use in the retinal screening decision process, this structure is able to accommodate independent and dependent ocular disease screening at a single building-level structure. All these considerations lead to the proposed framework effectively addressing the limitations identified through this research effort and therefore achieving the following three outcome improvements in relation to large-scale retinal screening: improved discriminative power, improved robustness, and improved scalability for population-based retina screening.

## 3. Proposed Method

**Figure 1:**
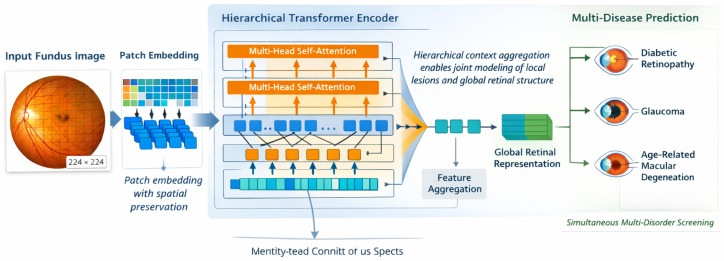
Overall architecture of the proposed transformer-based retinal screening framework, illustrating patch tokenization, hierarchical transformer encoding, feature aggregation, and multidisease prediction.

This section presents the proposed transformer-based framework for large-scale retinal image screening. The design emphasizes global contextual modeling, scalability to large and heterogeneous datasets, and suitability for multi-disease ophthalmic screening. This pipeline consists of the image preprocessing and tokenization, the transformer-based feature encoding module, the screening-oriented classification head, and the effective training strategy designed for large-scale retinal datasets. Figure 1 provides an overview of the overall framework in which the proposed pipeline transforms each fundus image into patch tokens and develops multi-level representations for simultaneous screening for diabetic retinopathy, glaucoma, and age-related macular degeneration.

### 3.1. Image Preprocessing and Patch Tokenization

Consider a multi-channel intensity representation of a color fundus image from a population-based screening program denoted as $I\in R^{H_{0}\times W_{0}\times C}$, where the height, $H_{0}$, width, $W_{0}$, and color channels, *C*, define a two-dimensional space that contains intensity values. Due to differences in hardware, illumination, and the method of capturing the image, each instance of *I* can have very different probabilities of generating based on large variability, such as inter-sample distributional shifts. If left unprocessed before representation learning occurs, these distributional shifts could negatively impact downstream representation learning. As a result, a standardized preprocessing pipeline is implemented to transform each instance of *I* into a normalized version so that they can now be statistically aligned with respect to one another and able to be tokenized in transformer-based architectures. The first step in this pipeline involves normalizing each channel of *I* based on their respective channel-wise mean/standard deviation computed over the training sample, which helps to remove variable amounts of light exposure when accounting for illumination in the images. In addition, let Ic(x,y) denote an intensity value of a specific channel at a specific pixel location (x,y) within image *I*. For each channel, normalization is achieved by subtracting the channel mean $\mu_{c}$ and dividing by the channel standard deviation $\sigma_{c}$, both computed over the training set. The normalized image, denoted by In, is defined as follows:

Let *I* denote a retinal fundus image acquired from a large-scale screening program, where $I\in R^{H_{0}\times W_{0}\times C}$ represents a multi-channel intensity function defined over a two-dimensional spatial domain with height $H_{0}$, width $W_{0}$, and *C* color channels. Due to variability in imaging devices, illumination conditions, and acquisition protocols, the raw image *I* exhibits significant inter-sample distributional shifts that can negatively impact downstream representation learning. To address this challenge, a standardized preprocessing pipeline is applied to transform *I* into a normalized representation that is statistically aligned across the dataset and suitable for transformer-based tokenization. The first preprocessing step performs channel-wise color normalization to reduce illumination-induced variations. Let $I_{c}\left(x,y\right)$ denote the intensity value of channel *c* at spatial location (x,y). For each channel, normalization is achieved by subtracting the channel mean $\mu_{c}$ and dividing by the channel standard deviation $\sigma_{c}$, both computed over the training set. The normalized image $I_{n}$ is defined as follows:

(1) $$I_{n}\left(x,y,c\right)=\frac{I\left(x,y,c\right)-\mu_{c}}{\sigma_{c}}$$

This channel-wise normalization operation standardizes each color channel to have approximately zero mean and unit variance, which improves numerical stability during optimization and reduces sensitivity to global intensity variations across images. Additionally, the normalized image as denoted by $I_{n}$ will keep the original spatial resolution but will have more statistical consistency for all of the images pooled together. The next step after successfully normalizing each of the three channels of the image is to implement a background mask used to eliminate all other regions besides the retina that do not provide useful diagnostic value. Let M(x,y) designate the binary mask for the field of view of the retina, where M(x,y)=1 indicates the pixel belongs to the retina’s field of view and M(x,y)=0 indicates otherwise. The resulting image from applying the binary mask to the normalized image is called $I_{m}$, which is generated by performing an element-wise multiplication on the two matrices.

(2) $$I_{m}\left(x,y,c\right)=I_{n}\left(x,y,c\right)M\left(x,y\right)$$

The use of the masking step guarantees that only relevant retinal information would be processed in any further stages using the transformer and would not enable attention to be allocated towards background artifacts. Once this is completed, the image $I_{m}$ is resized to a fixed image resolution H×W, producing a standardized input across all samples. Then the resized image will be broken down into non-overlapping square patches that make up the image, with the size of the patches being defined as *P*, and, for example, *H* and *W* being divisible by *P*; there will be a total of $N=\frac{HW}{P^{2}}$ patches in total. Each individual patch will be converted into a flattened vector $z_{i}\in R^{P^{2}C}$ according to the location of the patches within the image i∈{1,2,…,N}.

(3) $$z_{i}\in R^{P^{2}C}$$

While discarding explicit spatial ordering, flattened patch vectors capture localized appearance information about the retina. To project each patch into the same latent embedding space, a random linear projection is used. Define the embedding dimension *E*. The embedding of the patch will be mapped into a common latent ’embedding of the patch’ that has been made to use learnable linear projections, where $W_{p}\in R^{E\times P^{2}C}$. Finally, the embedded patch representation will be denoted as $e_{i}$; this is done as follows:

(4) $$e_{i}=W_{p}z_{i}$$

All patch embeddings will be the same dimensionally, *E*, in order for the transformer operations to continue. The embedding alone does not include the embedding of the patches relative to one another. Positional embeddings will help to encode spatial information back into the token embeddings so that we can use them to find similarity between the patches. We denote $p_{i}\in R^{E}$ as the learnable positional embedding for the *i*-th patch.

(5) $$t_{i}=e_{i}+p_{i}$$

The resulting token $t_{i}$ combines local visual content with explicit spatial context, enabling the transformer to maintain awareness of the retinal topology during global attention computation. The sequence of tokens $\left\{t_{1},t_{2},\ldots ,t_{N}\right\}$ produced at this stage forms the input to the transformer-based global feature encoding module, where long-range spatial dependencies across the retinal image are explicitly modeled.

### 3.2. Transformer-Based Global Feature Encoding

Let $T=\{t_{1},t_{2},\ldots ,t_{N}\}$ denote the sequence of patch tokens produced by the image preprocessing and patch tokenization stage, where each token $t_{i}\in R^{E}$ lies in an *E*-dimensional latent embedding space. The goal of the transformer-based global feature encoding module is to transform this initial token sequence into a context-aware representation in which each token captures both its local retinal characteristics and its relationships with all other spatial regions in the image. This global contextualization is essential for large-scale retinal screening, as diagnostically relevant patterns often span anatomically distant regions and cannot be reliably modeled using purely local feature extractors.

The transformer encoder is composed of *L* stacked encoding layers. Let $T^{\left(0\right)}=T$ denote the input token sequence, and let $T^{\left(l\right)}=\left\{t_{1}^{\left(l\right)},t_{2}^{\left(l\right)},\ldots ,t_{N}^{\left(l\right)}\right\}$ denote the output of the *l*-th transformer layer, where l∈{1,2,…,L}. Each layer refines the token representations by integrating information from the entire sequence through self-attention, thereby progressively enriching each token with global retinal context. Within each transformer layer, self-attention is computed by projecting the input tokens into three distinct representations referred to as queries, keys, and values. For layer *l*, let $W_{q}^{\left(l\right)}$, $W_{k}^{\left(l\right)}$, and $W_{v}^{\left(l\right)}$ denote learnable projection matrices. For each token $t_{i}^{\left(l-1\right)}$, the corresponding query, key, and value vectors are defined as follows:

(6) $$q_{i}^{\left(l\right)}=W_{q}^{\left(l\right)}t_{i}^{\left(l-1\right)}$$

(7) $$k_{i}^{\left(l\right)}=W_{k}^{\left(l\right)}t_{i}^{\left(l-1\right)}$$

(8) $$v_{i}^{\left(l\right)}=W_{v}^{\left(l\right)}t_{i}^{\left(l-1\right)}$$

Here, $q_{i}^{\left(l\right)}$, $k_{i}^{\left(l\right)}$, and $v_{i}^{\left(l\right)}$ encode how the *i*-th retinal patch attends to other patches, how it is attended by others, and what information it contributes to the aggregation process, respectively. These projections allow the transformer to compute pairwise interactions between all tokens in the sequence. The similarity between the *i*-th query and the *j*-th key is computed using a scaled dot-product operation. Let *d* denote the dimensionality of the query and key vectors. The attention coefficient $a_{ij}^{\left(l\right)}$ is defined as a normalized similarity score given by the following expression:

(9) $$a_{ij}^{\left(l\right)}=\frac{\exp\left(\frac{q_{i}^{\left(l\right)}\cdot k_{j}^{\left(l\right)}}{\sqrt{d}}\right)}{\sum_{m=1}^{N}\exp\left(\frac{q_{i}^{\left(l\right)}\cdot k_{m}^{\left(l\right)}}{\sqrt{d}}\right)}$$

This normalization ensures that $\sum_{j=1}^{N}a_{ij}^{\left(l\right)}=1$ for each query token *i*, allowing the model to distribute attention across all retinal regions in a probabilistic manner. The scaling factor $\sqrt{d}$ stabilizes gradient magnitudes during training and prevents numerical saturation of the exponential function. Using these attention coefficients, the self-attention output for token *i* at layer *l* is computed as a weighted aggregation of all value vectors in the sequence.

(10) $$s_{i}^{\left(l\right)}=\sum_{j=1}^{N}a_{ij}^{\left(l\right)}v_{j}^{\left(l\right)}$$

The vector $s_{i}^{\left(l\right)}$ is a globally informed embedding that allows distant retinal regions to directly influence the representation for token i with respect to the *i*-th retinal patch, thus allowing the transformer to model long-range spatial dependencies necessary for robust retinal screening. In order to create diverse representations, multiple attention heads act in parallel within the transformer. Each attention head utilizes different projection matrices and captures complementary relationships among retinal image tokens. Following this, all attention head output representations are concatenated and passed through to a linear transformation in order to generate an overall attention output for that layer. Once each attention head has provided its output, each token representation is transformed using position-wise non-linear transformations implemented via a feed-forward network. Let $W_{1}^{\left(l\right)}$ and $W_{2}^{\left(l\right)}$ be learnable matrices of the aforementioned transformation, and let ϕ(·) denote an element-wise, non-linear activation function. The feed-forward output for token i is defined as follows:

(11) $$f_{i}^{\left(l\right)}=W_{2}^{\left(l\right)}\phi \left(W_{1}^{\left(l\right)}s_{i}^{\left(l\right)}\right)$$

To preserve information across layers and ensure stable optimization in deep transformer stacks, residual connections are applied. The final output token at layer *l* is computed by adding the input token representation to the feed-forward output.

(12) $$t_{i}^{\left(l\right)}=t_{i}^{\left(l-1\right)}+f_{i}^{\left(l\right)}$$

**Figure 2:**
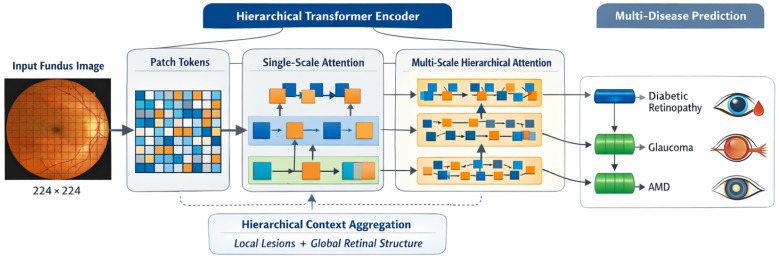
Detailed design of the hierarchical transformer encoder showing patch tokens, single-scale attention, multi-scale hierarchical attention, and the resulting global retinal representation used for multi-disease prediction.

Through the iterative application of these transformations across *L* layers, the transformer encoder produces a sequence of globally contextualized token embeddings that jointly encode local retinal appearance and long-range spatial dependencies. These representations form the basis for the hierarchical aggregation and screening-oriented prediction modules described in subsequent subsections. Figure 2 details the hierarchical transformer encoder, highlighting the transition from single-scale attention to multi-scale hierarchical attention for capturing both local lesions and global retinal structure.

### 3.3. Hierarchical Context Aggregation Strategy

Let $T^{\left(l\right)}=\left\{t_{1}^{\left(l\right)},t_{2}^{\left(l\right)},\ldots ,t_{N}^{\left(l\right)}\right\}$ denote the sequence of token representations produced by the *l*-th transformer layer, where each token $t_{i}^{\left(l\right)}\in R^{E}$ encodes retinal information at a specific level of abstraction. Global self-attention allows all tokens to communicate with every other token. However, if we use the same attention mechanism for all layers, we may use resources unnecessarily, and the result may be less sensitive to local pathological cues due to the change in scale. To overcome this problem, we adopted a hierarchical context aggregation model, where with each successive transformer layer the effective contextual window is increased for each token processed, so that fine-scale information can be gathered very early and emphasis is placed on global anatomical relationships at the deeper layers. In the lower layers of the transformer encoder, the focus of attention is on local neighborhoods, to capture fine-scale retinal features such as microaneurysms, bleeding and irregular blood vessels. Let $N_{i}^{\left(l\right)}$ be defined as the local neighborhood around token *i* at layer *l*, and consists of all of the tokens that are spatially adjacent to $t_{i}^{\left(l-1\right)}$. The restricted attention coefficients computed in the earlier layers of the transformer encoder will thus be created by restricting the normalization region of the attention function to $N_{i}^{\left(l\right)}$ only.

(13) $$a_{ij}^{\left(l\right)}=\frac{\exp\left(\frac{q_{i}^{\left(l\right)}\cdot k_{j}^{\left(l\right)}}{\sqrt{d}}\right)}{\sum_{m\in N_{i}^{\left(l\right)}}\exp\left(\frac{q_{i}^{\left(l\right)}\cdot k_{m}^{\left(l\right)}}{\sqrt{d}}\right)}$$

Here, $q_{i}^{\left(l\right)}$ and $k_{j}^{\left(l\right)}$ denote the query and key vectors associated with tokens *i* and *j* at layer *l*, and *d* refers to the dimension of the attention subspace for the transformer in which the tokens from that input sequence reside. For early-layer attention to focus primarily on local consistency, and to diminish the influence of noise from remote sources, we restrict our attention to nearby tokens in the context to which the respective token is attempting to attend $N_{i}^{\left(l\right)}$. This is particularly useful in terms of identifying subtle, pathological patterns that exist within highly constrained spatial dimensions. As the transformer gains depth, the specification of context becomes increasingly more relaxed, thereby allowing for increased interaction among the broader context. We define the layer-dependent expansion coefficient $\alpha^{\left(l\right)}\in \left[0,1\right]$, where smaller values exist closer to the center of the range, representing an expansion of attention across a small region, and larger values represent an expansion of attention across a much larger region regarding its global context. The effective range of attention for layer *l* therefore consists of a weighted combination of the component forms of, and defined by, local and global attention mechanisms.

(14) $${\tilde{a}}_{ij}^{\left(l\right)}=\alpha^{\left(l\right)}a_{ij,global}^{\left(l\right)}+\left(1-\alpha^{\left(l\right)}\right)a_{ij,local}^{\left(l\right)}$$

In this formulation, $a_{ij,local}^{\left(l\right)}$ is computed using the restricted neighborhood $N_{i}^{\left(l\right)}$, $a_{ij,global}^{\left(l\right)}$ refers to all tokens from {1,2,…,N}. The term $\alpha^{\left(l\right)}$, or the aggregation coefficient, grows monotonically with layer number *l*, thus allowing for a continuous transition of the aggregation from local to global features across the layers. Both $a_{ij,\mathrm{local}}^{\left(l\right)}$ and $a_{ij,\mathrm{global}}^{\left(l\right)}$ are individually normalized via softmax such that $\sum_{j=1}^{N}a_{ij,\mathrm{local}}^{\left(l\right)}=1$ and $\sum_{j=1}^{N}a_{ij,\mathrm{global}}^{\left(l\right)}=1$. Since the aggregation coefficient satisfies $\alpha^{\left(l\right)}\in \left[0,1\right]$, the mixed attention in (14) forms a convex combination of two probability distributions. Therefore, the combined coefficients also satisfy

(15) $$\sum_{j=1}^{N}{\tilde{a}}_{ij}^{\left(l\right)}=1,$$

and no additional renormalization step is required. This preserves the probabilistic interpretation of attention weights across all hierarchy levels. Aggregated attention coefficients are used to compute context-enhanced token reverberations at layer *l* by taking a weighted sum over the value vectors. In particular, let $v_{j}^{\left(l\right)}$ indicate the value vector associated with token *j* in layer *l*. The representation of token *i* in layer *l*, denoted as $s_{i}^{\left(l\right)}$, is computed according as

(16) $$s_{i}^{\left(l\right)}=\sum_{j=1}^{N}{\tilde{a}}_{ij}^{\left(l\right)}v_{j}^{\left(l\right)}$$

A hierarchical aggregation allows each of its tokens to propagate through the hierarchy and to build progressively upon information about more distant regions on the retina. This way, the first representations maintain the same sensitivity to localized pathology, while each consecutive abstraction encodes information about a larger global and anatomical context and larger lesions located in the overall distribution. As a means of enhancing the hierarchical abstract of representations, intermediate token representations are pooled together at certain depths of the hierarchy to form multi-scale contextual embeddings. Let $l_{1},l_{2},\ldots ,l_{K}$ denote the layers of transformers selected at varying abstraction levels. To create a pooled representation $h^{\left(k\right)}$ at abstraction level *k*, token embeddings from layer $l_{k}$ will be averaged together.

(17) $$h^{\left(k\right)}=\frac{1}{N}\sum_{i=1}^{N}t_{i}^{\left(l_{k}\right)}$$

The complementary contextual information that can be shared would range from fine-scale feature capture to global retinas. It is important to clarify that the proposed hierarchy is realized through progressive attention-range expansion and multi-depth fusion, rather than through a token-resolution pyramid. In particular, models such as Swin transformer employ a hierarchical design primarily by reducing spatial resolution via patch merging and performing attention within shifted local windows, which yields a stage-wise feature pyramid. In contrast, our formulation keeps patch tokens as the common representation while explicitly controlling the effective receptive field of attention across layers. This is achieved by combining a locally normalized neighborhood attention with a globally normalized full-sequence attention via a layer-dependent mixing coefficient, where $\alpha^{\left(l\right)}$ increases with depth to provide a continuous transition from lesion-sensitive local interactions to global anatomical context. Furthermore, instead of relying only on the final layer representation, we explicitly pool intermediate token embeddings from selected depths and fuse them using learnable weights $\left\{\beta_{k}\right\}$, yielding a single global retinal representation that jointly encodes fine-grained pathology and large-scale retinal structure for screening. *h* is then formed through aggregation of all the embeddings at all selected levels.

(18) $$h=\sum_{k=1}^{K}\beta_{k}h^{\left(k\right)}$$

In this case, the variable called $\beta_{k}$ is simply a learnable scalar weight satisfying $\sum_{k=1}^{K}\beta_{k}=1$ to adaptively balance the contribution of all the different contextual scales and to produce a representation of the pathology being modeled. This hierarchical approach for aggregating context allows for improved discrimination between different disease categories that are visually similar by jointly modeling both local pathology and global anatomy, while also reducing the sensitivity to local noise and imaging artifacts typically present in large population scale retinal screening datasets.

The proposed approach differs not only in terms of mechanism but also in terms of objective from current approaches to hierarchical vision transformers. For example, the Swin transformer and other multi-scale ViT architectures establish a hierarchy primarily by progressively down-sampling tokens and employing window-based local attention to construct a spatial feature pyramid. The proposed model, on the other hand, does not change the representation of tokens but instead introduces a progressive scheduling mechanism for attention range, where the effective receptive field of attention is increased progressively across layers by means of learnable coefficients $\alpha^{\left(l\right)}$. In addition, instead of relying only on the final stage representation, the proposed model explicitly fuses contextual embeddings from multiple depths using learnable weights $\left\{\beta_{k}\right\}$ in order to jointly model fine-grained lesions with associated anatomical structure within a single representation—making it especially well-suited for large-scale screening of the retina, where subtle localized pathology must be detected together with widespread structural context without excessive spatial down-sampling.

### 3.4. Screening-Oriented Prediction Head

Denote the final hierarchical rendition (i.e., global retinal representation) produced by the transformer encoder and the hierarchical context aggregation mechanism as $h\in R^{E}$ where *E* is the dimension of the embedding space for the tokens mapped into this representation. The screening-oriented prediction head’s goal is to map this global retinal representation to clinically relevant screening outputs with high computational efficiency for populations of the size that would be required for population-wide use, unlike general classification heads, the proposed prediction head has been explicitly formulated to allow both single-disease and multi-disease screening paradigms under a single unified mathematical framework, thus providing flexibility in how they may be deployed across different types of ophthalmic screening programs. After applying a linear transformation of the global representation of *h* to obtain a task specific latent representation, let *D* represent the dimensionality of this intermediate screening space; let $W_{s}\in R^{D\times E}$ represent the learnable projection matrix. Then the screening feature vector $r\in R^{D}$ is defined as

(19) $$r=W_{s}h$$

The transformation processes information in a hierarchical structure into a compact representation, which maintains all the clinically relevant characteristics while at the same time reduces redundancy and decreases the costs associated with inferring, as well as uses less memory when performing large-scale population screening activities. When screening for a single disease, the goal of the model is to predict whether or not that condition exists with y∈{0,1} representing the binary disease label of the image, where a value of y=1 indicates that the disease is present. The predicted probability of disease $\hat{y}$ is calculated by applying a sigmoid activation to a scalar logit derived from the hierarchical representation determined by the preceding process *r*, given certain weights $w_{c}\in R^{D}$ that correspond to each image in the dataset.

(20) $$\hat{y}=\frac{1}{1+\exp(-w_{c}\cdot r)}$$

Threshold-based decisions, which are often used in clinical settings to balance sensitivity and specificity, can be made with the help of this probabilistic output. The binary cross-entropy loss function is the common loss function used when you are screening patients for only one disease. This function is used to determine how far off your estimate is based on the actual outcome, by comparing the predicted probability of having or not having the disease against what actually happened.

(21) $$L_{\mathrm{single}}=-\left[y\log\left(\hat{y}\right)+\left(1-y\right)\log\left(1-\hat{y}\right)\right]$$

The multi-disease screening problem is about predicting multiple ophthalmological diseases at once based on one retinal image. We define *C* to be the number of diseases, and $y=[y_{1},y_{2},\ldots ,y_{C}]$ as a vector of true multi-labels indicating if a patient has a disease ($y_{c}=1$) or does not have a disease ($y_{c}=0$) at each category *c*. The prediction head will produce a logit vector from the retinal image *r* using a linear transformation.

(22) $${\hat{y}}_{c}=\frac{1}{1+\exp(-w_{c}\cdot r)},\;\;\;\;c=1,2,\ldots ,C$$

Each disease classifier, denoted by $w_{c}\in R^{D}$, corresponds to a different disease in such a way that each classifier can be modeled independently. This model avoids the assumption of mutual exclusivity and thus is appropriate for the task of retinal screening, as there may be multiple diseases in one image. For doing a multi-disease screening, the loss function is defined as the sum of binary cross-entropy losses for each of the disease categories.

(23) $$L_{\mathrm{multi}}=-\sum_{c=1}^{C}\left[y_{c}\log\left({\hat{y}}_{c}\right)+\left(1-y_{c}\right)\log\left(1-{\hat{y}}_{c}\right)\right]$$

Each disease should be predicted accurately by separate predictions while allowing features to be shared across all diseases via a common representation *r*. For use at the population level, the prediction head will be kept as light as possible by using only linear transformations and element-wise non-linearities as part of a design that results in low latency and low computational costs. This design will allow for the rapid evaluation of many patients with little to no decrease in diagnostic accuracy. The outputs of the prediction head will be used as the final results of the screening and will be used to identify patients who should be triaged in large-scale retinal screening programs.

### 3.5. Training Strategy and Optimization

The supervised training dataset $D={\left\{\left(I_{n}^{\left(i\right)},y^{\left(i\right)}\right)\right\}}_{i=1}^{M}$ is composed of *M* samples, where the image portion corresponds to the *M*-th image being denoted as $I_{n}^{\left(i\right)}$ and the corresponding annotation being denoted as $y^{\left(i\right)}$ for the *M*th image as well. Both the image and the corresponding annotation can be either a single disease label or a vector of multi-dimensional/multi-label diseases. The training objective is to learn the set of model parameters Θ that minimize the expected prediction error over the large-scale data distribution. During training, each input image $I_{n}^{\left(i\right)}$ is passed through the preprocessing, patch tokenization, transformer-based global encoding, and hierarchical context aggregation modules, resulting in a global representation $h^{\left(i\right)}$. The screening-oriented prediction head then produces an output ${\hat{y}}^{\left(i\right)}$ that approximates the true label $y^{\left(i\right)}$. The learning process is formulated as a risk minimization problem, where the empirical loss over the dataset is minimized.

(24) $$L\left(\Theta \right)=\frac{1}{M}\sum_{i=1}^{M}\ell \left(y^{\left(i\right)},{\hat{y}}^{\left(i\right)}\right)$$

Here, ℓ(·,·) denotes a task-specific loss function that quantifies the discrepancy between the predicted output and the ground-truth label. For single-label screening tasks, where each image is associated with a single disease category, the loss function is defined using categorical cross-entropy. Let $y^{\left(i\right)}\in \left\{0,1\right\}$ denote the binary label and let ${\hat{y}}^{\left(i\right)}\in \left(0,1\right)$ denote the predicted probability.

(25) $$\ell_{\mathrm{single}}^{\left(i\right)}=-\left[y^{\left(i\right)}\log\left({\hat{y}}^{\left(i\right)}\right)+\left(1-y^{\left(i\right)}\right)\log\left(1-{\hat{y}}^{\left(i\right)}\right)\right]$$

For multi-disease screening tasks, each image may exhibit multiple co-occurring pathologies. In this case, the label $y^{\left(i\right)}=\left[y_{1}^{\left(i\right)},y_{2}^{\left(i\right)},\ldots ,y_{C}^{\left(i\right)}\right]$ is a binary vector of length *C*, where *C* denotes the number of disease categories. The corresponding predicted output is ${\hat{y}}^{\left(i\right)}=\left[{\hat{y}}_{1}^{\left(i\right)},{\hat{y}}_{2}^{\left(i\right)},\ldots ,{\hat{y}}_{C}^{\left(i\right)}\right]$. The multi-label loss is defined as the sum of binary cross-entropy terms across all disease categories.

(26) $$\ell_{\mathrm{multi}}^{\left(i\right)}=-\sum_{c=1}^{C}\left[y_{c}^{\left(i\right)}\log\left({\hat{y}}_{c}^{\left(i\right)}\right)+\left(1-y_{c}^{\left(i\right)}\right)\log\left(1-{\hat{y}}_{c}^{\left(i\right)}\right)\right]$$

In order to enhance generalizing across various devices and varied populations of imaging devices, augmentation of the initial training dataset would occur. The random application of random color variations, contrast modifications and spatial transformations to an input image will result in new versions of the original training imaging sample known as ${\tilde{I}}_{n}^{\left(i\right)}$ being generated from effects produced using the stochastic data augmentation operator denoted as A(·).

(27) $${\tilde{I}}_{n}^{\left(i\right)}=A\left(I_{n}^{\left(i\right)}\right)$$

The process of augmenting training samples will implicitly expand the support of your training distribution and help you train a model that learns invariant representations that can withstand any sort of variability that might arise as a result of how the data was obtained. Thus, the loss function will be based on augmented sample data, which will help prevent overfitting to data particular artifacts in the training set. All model parameters will be adjusted through a method using adaptive gradient based methods. Let $Theta_{t}$ represent your Parameters at iteration *i* and let $\nabla_{\Theta }L_{t}$ be the gradient of the loss with respect to $\Theta_{t}$, the update rule for your model parameter is as shown below:

(28) $$\Theta_{t+1}=\Theta_{t}-\eta_{t}\nabla_{\Theta }L_{t}$$

Learning rate at iteration t is indicated by $\eta_{t}$. To improve the stability of convergence using a learning rate scheduling technique, with decreasing schedule values, an initial learning rate $\eta_{0}$ will be given a decay of type γ∈(0,1). The scheduled learning rate is given below:

(29) $$\eta_{t}=\eta_{0}\gamma^{t}$$

With rapid initial learning and then gradually refining that initial learning through more finely grained convergences at the later stages of training, this optimization approach achieves stable training behavior and good generalization performance for large-scale retinal image screening scenarios by combining task specific loss functions with stochastic data augmentations and adaptive optimization with learning rate scheduling.

## 4. Experimental Results

### 4.1. Datasets and Experimental Protocol

This subsection gives a summary of the datasets used for population-level retinal screening and defines a common protocol for conducting experiments that will lead to equitable and reproducible results among different methods. Two large public retinal fundus datasets were used in the experiments; the two datasets represent complementary approaches to screening: screening for one disease (single-disease) and screening for multiple diseases (multi-disease). Each of the models was evaluated using the same data splits, preprocessing pipelines, and training procedures in order to avoid introducing bias associated with any specific dataset and to provide an opportunity to compare results with existing baseline models.

The first dataset is intended for large-scale screening for a single disease and is concerned with the evaluation of diabetic retinopathy severity; on the other hand, the second dataset supports multi-disease screening of several different types of ophthalmic diseases. The images contained within each dataset are split into training, validation and test subsets at the patient level in order to limit potential information leakage. The same procedures as those recommended in the forthcoming methodology are utilized with regard to preprocessing and include color normalization, masking of the background, standardization of resolution and tokenizing of patches; these steps are employed consistently across all tests. Benchmarking is performed using fixed splits so as to create reproducible results across multiple runs. An overview of the datasets, including: the size of each dataset; the total number of disease categories; the nomenclature used to designate the type of disease screening; and the strategy employed to achieve data partitioning, is shown in Table 2.

In addition to the size of the dataset, class imbalance is a major consideration for retinal screening work. This is especially true for large population level datasets with a high number of normal cases. For the purpose of transparency regarding both disease and screening prevalence, we provide a summary of class distribution statistics for both datasets. Class distribution statistics are also intended to guide the selection and interpretation of evaluation metrics, especially for diseases in which sensitivity is important. In the report of class distribution in Table 3, the class distributions show that class imbalances are a defining characteristic of actual screening situations. The existing class imbalances require the use of robust evaluation metrics and repeated experimental evaluations in order to generate reliable performance estimates. To mitigate the effect of severe class imbalance typical in population screening, evaluation emphasizes macro-averaged metrics and per-disease sensitivity analysis. The consistent performance across minority disease categories in RFMiD suggests that the proposed hierarchical aggregation helps preserve discriminative features even for underrepresented conditions.

All experiments across datasets use a common protocol; model training is performed using the same data preprocessing procedure, the same patch sizes and the same optimization configuration; each experiment that utilizes the proposed framework is completed three times with different random seeds. All results in the following sections are reported as mean pm for the proposed approach. This common protocol has been employed in order to achieve robustness and reproducibility, as well as to ensure that all large-scale retinal screening benchmarks can be directly compared. For fair comparison, all baseline models reported in the subsequent experimental tables are re-implemented and evaluated under the unified protocol described above, including identical data splits, preprocessing, training schedules, and evaluation metrics. Consequently, minor numerical differences may exist between the literature-reported results and the controlled experimental results.

A consistent use of a fixed input resolution and patch-based tokenization allows for processing of retinal fundus images in a uniform manner across different datasets. The selected depth of transformers, embedding dimension and attention configuration provide sufficient global context to be obtained at a reasonable training and inference cost. All training was conducted using mini-batch optimization with adaptive learning rates, and early stopping based on validation performance to minimize the possibility of overfitting. The core architecture and training hyperparameter configuration used in all experiments are shown in Table 4.

As well as differences in core architectural parameters, the time to train and the convergence behavior of the training process for different datasets can also be distinguished based on size and complexity of the labeled dataset. The EyePACS experiments have considerably longer training times relative to RFMiD experiments, with EyePACS datasets requiring longer training times due to the greater number of images compared to RFMiD datasets and the class imbalance resulting from the use of large scale populations to screen for disease, while RFMiD experiments have a shorter time until convergence because the supervision labels are more complex since the datasets are multi-label. The computational requirements provided are as follows: the epoch-based number of training epochs and the total training time for the dataset. Table 5 outlines the measured training efficiency statistics for each dataset based on identical hardware configuration. These results allow for evaluation of the proposed framework’s potential scalability whilst providing a context for assessing whether the proposed framework can be effectively used for large scale screening projects.

To provide consistent performance measurements during experimentation, all trials are run on dedicated GPU-based compute environments. Inference efficiency is the most important factor in determining how effective large population screening systems will be because they may require processing hundreds or thousands of images per day. Inference latency and throughput are measured according to both batch mode and single image mode. A summary of the hardware and software environments used in all experiments can be found in Table 6 and provides details on the computational resources available, allowing for independent and reproducible implementations of each of the experiments.

A set of methods will also be developed to evaluate how to apply the best practices of population-based eye examinations, using a range of metrics that reflect the needs for population-wide retina screening, so that single disease versus multiple disease evaluations can be fairly compared. Screening performance is assessed using accuracy, which captures the proportion of correctly classified samples, while macro-averaged F1 score is reported to account for class imbalance by equally weighting all disease categories. Sensitivity and specificity are included to reflect clinically relevant trade-offs between false negatives and false positives, which are critical in screening applications where missed diagnoses carry high risk. In addition, the area under the receiver operating characteristic curve is reported to evaluate the model’s ability to rank diseased and non-diseased cases across varying decision thresholds. For multi-disease screening, metrics are computed independently for each disease and then aggregated using macro-averaging to avoid bias toward prevalent conditions. All metrics are computed on held-out test sets using fixed thresholds determined on validation data, ensuring consistent and unbiased evaluation across datasets and methods.

The architecture put forth is based on an architecture to ensure that it is scalable as stated in Table 5. The training process has been successful to produce converged results on both EyePACS and RFMiD datasets within a reasonable amount of time as shown in the previous Table 6. In addition, the hierarchical network’s moderate level of computational complexity is attributed to its fixed patch token lightweight screening modality, both of which provide a means to manage memory requirements and inference times. From an implementation perspective, the use of FP16 inference and batching processing allows for efficient, large scale screening implementations. While we have performed training using high-performance graphics processing unit hardware, there is a much less demanding computational resource requirement for the inference side of the network; therefore optimizations can be made through model compression and hardware-specific acceleration should there be any such need.

All experiments were repeated three times with different random seeds, and results are reported as mean ± standard deviation. The relatively small variance across runs indicates stable optimization behavior and consistent model performance, supporting the statistical reliability of the proposed framework. This initial evaluation shows that the model is robust regardless of the distribution of disease in the two public datasets and their respective screening contexts. It is not a replacement for eventual prospective clinical validation; however, because EyePACS and RFMiD have similar performance, both demonstrate promise for cross-domain generalization. Representative CNN and transformer baselines were selected to provide controlled and reproducible comparisons under a unified training protocol, enabling fair assessment of the proposed hierarchical design.

### 4.2. Single-Disease Screening Performance

Single-disease screening performance is evaluated on the EyePACS dataset, focusing on diabetic retinopathy detection and severity grading under a clinically realistic screening setup. Table 7 shows that the proposed method achieves the strongest overall performance for binary diabetic retinopathy screening on the EyePACS dataset, consistently outperforming both CNN- and transformer-based baselines across all evaluated metrics. Our model surpasses classical CNNs such as VGG-16, ResNet-50, and InceptionV3 regarding accuracy and AUROC, demonstrating increased ability to distinguish between classes. In addition, it achieves the highest sensitivity at 91.1%, which is essential when performing a large number of screenings to reduce false negative cases of diabetic retinopathy while still obtaining high and clinically acceptable specificity at 87.9%. While the proposed framework demonstrates significant enhancements on several measurement criteria, the overall AUROC gain compared to strong transformer-based systems has not been drastic. Therefore, since all improvements were measured in a controlled comparative experiment, any improvements seen could be taken as being similar to previously established levels of improvement and therefore do not provide strong evidence that one approach is superior to another for use in practicing medicine.

Table 8 provides an in-depth per-disease analysis of screening by severity of diabetic retinopathy in EyePACS to evaluate the clinical validity of the model beyond just aggregate metrics. The results show that the screening system has the best accuracy, sensitivity, and specificity for the no dr category, meaning it will be able to easily identify healthy patients and exclude non-pathological patients from the screening process. In contrast, performance on mild and moderate dr, representing early-stage and diagnostically difficult-to-diagnose stages of disease, is characterized by lower values for F1-score and sensitivity than the other categories because of the inherent challenge in visually identifying subtle lesions and the increased difficulty associated with identifying early-stage diseases. On the other hand, severe and proliferative dr both show consistently improved performance, characterized by higher F1-score and sensitivity values, which demonstrates a high degree of ability of the model to detect more advanced pathological conditions that urgently require clinical referral.

The performance of the various approaches used for diabetic retinopathy severity classification on the EyePACS dataset is summarized in Table 9. Each approach exhibits different degrees of success based on the use of CNN as opposed to transformer and proposed methodologies. The traditional CNN approaches demonstrate low levels of consistency with respect to grading, as evidenced by moderate levels of accuracy and macro-F1 score. This grading inconsistency is further evidenced by their inability to accurately identify and distinguish between adjacent severity levels consistently. The use of transformers added a significant performance benefit compared to CNN models for demarcating the difference between severity ratings due to the global context modeling and image classification capabilities associated with transformers. This is reflected in the substantially better performance demonstrated by the transformer baseline across both macro-F1 and accuracy score metrics. The proposed methodology produces the best overall performance of any methodology evaluated, achieving both the highest accuracy and macro-F1 score based on a more equitable and stable distribution of people through each of five severity grades. Additionally, the increase in the proposed methodology’s sensitivity and AUROC demonstrates improved rates for correct recognition of clinically significant moderate and severe cases, while the high specificity demonstrates accurate classifications of different stages of disease.

The proposed model has a good balance of sensitivity and specificity across datasets, which is critical for the large-scale screening environment in which missed diagnoses and unnecessary referrals must both be minimized. The thresholds used for operation were selected on validation data to simulate behavior typical of clinical screening. Reducing the number of false negative screenings is very important to screening. Because of the greater sensitivity that the proposed approach offers, fewer patients will be missed and those pathological cases identified. However, formal false-negative risk modeling of prospective patient cohorts is still an important area of study for future clinical validation.

### 4.3. Multi-Disease Screening Performance

The performance of multi-disease screening is assessed based on data from the RFMiD dataset, which serves as a realistic population-sized dataset to model screening scenarios for multiple co-occurring ophthalmic diseases from a single retinal fundus image. The evaluation focuses on how well different models can detect multiple types of diseases at the same time and/or remain consistent when testing for both commonly and rarely occurring diseases. Overall multi-disease screening performance results based on the RFMiD dataset are provided in Table 10, which allows for a comprehensive evaluation of the robustness of the model in a multi-label clinical screening setting. Of note, DenseNet, as a CNN-based baseline, provides consistently greater balanced performance than both ResNet-50 and MobileNetV3, which can be observed in its higher macro-F1 and sensitivity values. Furthermore, utilizing the Swin transformer for transformer-based modeling provides improved disease classification compared to CNN-based models. In addition to outperforming all competing methods, our method exhibits improved accuracy and macro-F1 score, indicating consistent, reliable, and balanced disease classifications overall as well as across various disease classes in the face of the pre-existing class imbalance. Moreover, our method demonstrates greater sensitivity, with respect to pathological cases and missed diagnosis reduction, given a higher specificity for excluding irrelevant disease classes than all other competing methods.

The performance of the RFMiD dataset’s per-disease screening is presented in Table 11. This table provides detailed information on how well the models performed for different ophthalmic conditions that have a variety of pathology and prevalence. The proposed method shows the most accurate results and has the most balanced F1-scores across all three types of disease: vascular, structural, and degenerative. In addition, the methods showed improvements in sensitivity for clinically important diseases such as diabetic retinopathy, glaucoma, and macular degeneration, which is critical for improving missed diagnoses in population-based screenings. Furthermore, the specificity of the proposed method shows that it can effectively exclude patients who do not have the disease in question, regardless of how likely those patients are to have it.

### 4.4. Comparison with Baseline Methods

A large population-based study comparing the proposed framework against several classic transformer-based models for retinal imaging screening, including our approach, will be described and compared in order to analyze the overall capability of both systems within distinct clinical environment levels. Within the single-disease vs. multiple-disease scenarios evaluated across the entire benchmark of clinical environments, Table 12 illustrates the same performance output of the proposed hierarchical transformer as it does for all popular CNN-based models, demonstrating consistent and increasing overall performance due to increased representational capability for all traditional CNN architectures, while the global contextual nature of the vision transformer allows for an even higher level of accuracy and AUROC performance than traditional architectures. Thus, the proposed methodology consistently performs at or above each evaluation metric, achieving both maximum accuracy and AUROC along with maximum improvement in the sensitivity of screening for accurately identifying if an individual has DR, which is critical to reducing missed cases of DR in screening environments, and high specificity, indicating accuracy between true and false positive test results.

The proposed framework’s performance is compared against representative baselines from both CNN and transformer-based models for multi-disease screening using the RFMiD dataset in Table 13, which represents a difficult, multi-label clinical situation where diseases frequently occur together. In CNN architectures, DenseNet has better-balanced performance when evaluated with ResNet-50 and MobileNetV3. However, the Swin transformer improves on both of those CNN architectures with respect to accuracy and macro-F1 score because it takes advantage of hierarchical context for more accurately representing the relationship between diseases. The proposed framework routinely outperforms all benchmarks for every evaluation metric—achieving the highest levels of accuracy, macro-F1 score, sensitivity, and specificity across the board. The significant increase in macro-F1 score shows that the proposed framework provides a lower variance in recognizing each disease category even with significant class imbalance, while the increased sensitivity demonstrates that the proposed framework has greater capacity for accurately identifying co-occurring disease conditions.

### 4.5. Ablation Study

**Figure 3:**
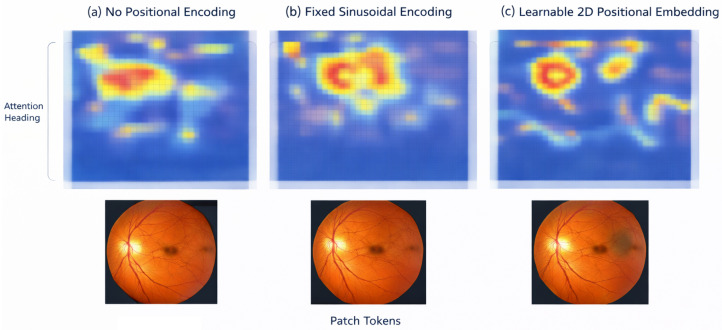
Qualitative effect of positional encoding on attention patterns.

**Figure 4:**
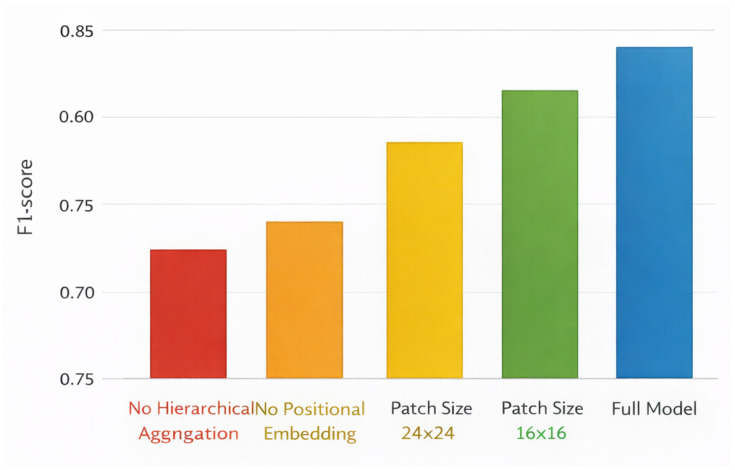
Ablation study summary illustrating performance degradation after removing hierarchical aggregation or positional encoding, and improvements obtained with optimal patch configuration and the full model.

Ablation analysis is used to quantify contributions from architectural and operational factors of the proposed method and the overall design decisions made in relation to large-scale retinal screening. The results are obtained over the EyePACS and RFMiD datasets and thus show consistent results for both single and multi-disease conditions. The results of ablation experiments evaluating the effects of hierarchical context aggregation and positional embeddings for both EyePACS and RFMiD datasets are included in Table 14. Ablating hierarchical context aggregation produced the greatest negative impacts on accuracy, macro-F1 score, and AUROC across all datasets, indicating that the ability to model context across multiple spatial scales is essential for adequately representing both local and global features of the retina. The ablation of positional embeddings also produced consistent negative impacts on performance, suggesting that explicit representations of spatial information are critical to produce robust learned representations of retinas. Additionally, performance on both datasets indicates that hierarchical and positional embeddings provide complementary information, indicating the need to include both for maximum predictive performance in screening. The accompanying visual content provided in Figure 3 and Figure 4 support both of these conclusions through the visualization of degraded attention localization and decreased overall screening robustness with the removal of each architectural component.

**Figure 5:**
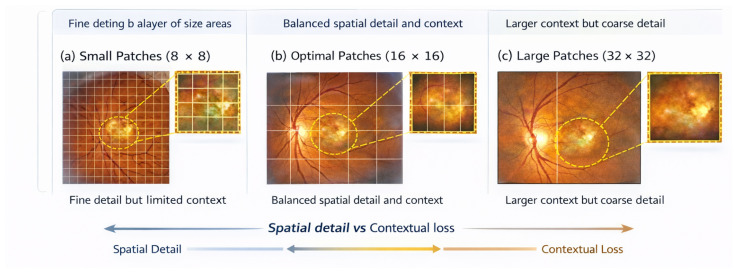
Patch size selection trade-off for retinal screening: smaller patches retain spatial detail, whereas larger patches provide broader context with increased contextual loss.

In Table 15, the impact of the choice of patch size on screening performance for the two EyePACS and RFMiD datasets is analyzed with respect to their local detail preservation and global context representation. That is, very small patches of 8×8 have the ability to capture the fine detail of lesions but show a slight decrease in accuracy and macro-F1 score due to increased sensitivity to other types of noise and a higher number of tokens. On the other end of the spectrum, very large patches of 32×32 produced a drop in performance across all metrics, indicating that critical spatial and pathological information was lost, thereby producing a faulty screening process. Intermediate-sized patches of 16×16 produced the most consistent performance across the two datasets and produced the highest accuracy, macro-F1 score and AUROC, at the same time demonstrating generalizability in both single-disease screening and multi-disease screening scenarios. Therefore, results in this table substantiate that the patch size selected is a well-balanced choice between local representation of lesions and global contextualized understanding of lesions, a sentiment that is visually displayed in Figure 5.

The impact of transformer encoder depth on screening performance for the datasets in EyePACS and RFMiD has been illustrated by an ablation study as depicted in Table 16. Fewer layers have reduced accuracy, macro-F1 score and AUROC, thus indicating limited capacity to capture either long-range dependencies or more global context of the retina. Performance is improved substantially across both datasets when increasing depth to 12 layers, which allows for better hierarchical feature learning and context aggregation. Performance benefits of any significant kind accrued during an increase in depth beyond 12 layers are negligible, resulting in diminishing returns of representational benefit as a result of increased computational complexity.

The summary in Table 17 details the impact of selecting the embedding dimensions used to screen patients across both EyePACS and RFMiD datasets. When using lower embedding dimensions, there was a notable decrease in the screening performance metrics, thus indicating that lower dimensions were not sufficient for capturing complex patterns found in retinal images. By increasing the embedding dimension to 768, there was a significant increase in every screening performance metric on both datasets, indicating use of a more expressive and discriminative feature space. Increasing the embedding dimension to 1024 yielded statistical improvements; however, these results demonstrate diminishing returns for the additional resource cost associated with the larger embedding dimension.

### 4.6. Computational Efficiency and Scalability Analysis

Efficient computation is a key requirement of retinal screening systems used at the population level. These screening systems require the processing of large amounts of fundus images within a short period of time, with a limited amount of hardware available to do so. This section will evaluate three main aspects of retinal screening system efficiency: model complexity, inference latency, and throughput. A comparison of model complexity in terms of the number of parameters, number of floating-point operations (FLOPs), and the amount of memory used is shown in Table 18 for a range of retinal screening system architectures. Classical CNN models display opposing profiles of efficiency; for example, VGG-16 has a very high number of parameters and a large memory footprint, while ResNet-50 and InceptionV3 achieve more efficient designs by being deeper while providing comparable or better predictive capabilities as compared to VGG-16. The vision transformer has a much higher computational and memory requirement than both CNNs due to the requirement of using global self-attention, making it difficult to deploy the vision transformer in resource-constrained retina screening environments. In contrast, the proposed hierarchical transformer displays a well-balanced profile with respect to complexity, requiring far fewer parameters and FLOPs than the vision transformer and providing a higher representational capacity than the baseline compact CNNs in this study.

**Figure 6:**
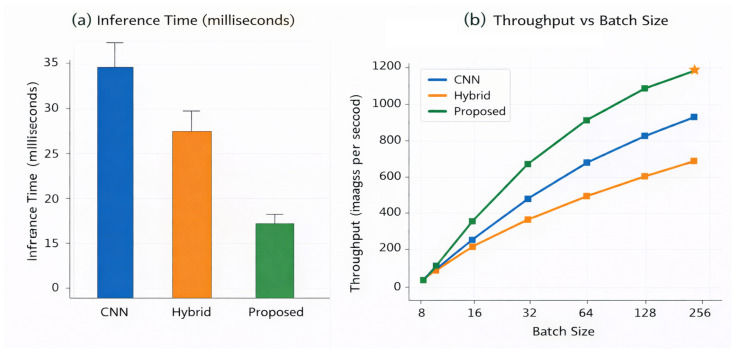
Computational efficiency comparison: (**a**) inference time and (**b**) throughput versus batch size for cnn, hybrid, and the proposed approach.

**Figure 7:**
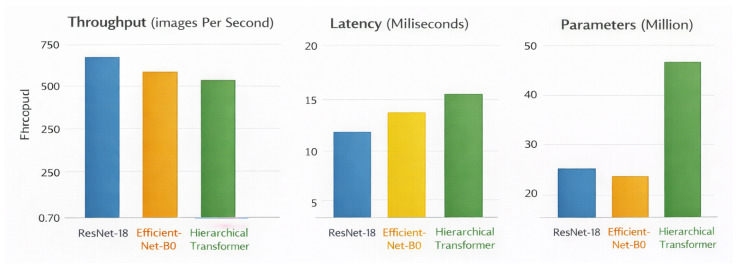
Comparison of inference efficiency across ResNet-18, EfficientNet-B0, and the proposed hierarchical transformer in terms of throughput, latency, and parameter count.

The inference latency of different models running under single image screening conditions, which is indicative of the slowest potential deployment scenarios in a clinical pipeline, is shown in Table 19. The amount of latency seen for most of the CNN models was primarily due to the compact structure of the ResNet-50 model, which had the least amount of latency, whereas the larger VGG-16 and InceptionV3 models had longer inference times due to their greater number of parameters and thus greater levels of computational complexity. As expected, the vision transformer has the greatest amount of latency, as global self-attention operations are very costly to process in terms of time. The proposed approach has a good latency-performance trade-off; the proposed approach has a substantially faster inference time compared to the vision transformer while, at the same time, having a comparable level of performance as compared to the most efficient CNN models. This level of latency is sufficient for real-time screening of patients within a high-throughput clinical workflow, which is supported by the throughput and efficiency comparison results shown in Figure 6 and Figure 7.

The throughput comparison under batch inference conditions is demonstrated in Table 20, which is representative of potential deployment scenarios such as large centralized screening servers and large-scale population screening programs. The ResNet-50 CNN baseline demonstrates the highest throughput of all three CNN baselines because of structural efficiency in architecture. In contrast, VGG-16 and InceptionV3 demonstrate much lower throughput due to the fact that they have a much higher demand for computation and memory resources than ResNet-50. The vision transformer exhibits the lowest throughput due to the inherent overhead associated with global self-attention when batching a large number of items together; therefore, the proposed framework provides a consistently higher throughput than the vision transformer while still achieving similar processing speeds for all three efficient CNN models, demonstrating the proposed framework’s viability as a well-balanced trade-off between computation efficiency and representational capacity.

Table 21 presents the scalability analysis for different models applied to larger batches of images and how each model performs under increasing workloads in terms of latency. All architectures see an increase in their latency as the batch sizes increase; however, the magnitude of the increase differs greatly by architecture. The VGG-16 architecture has the highest growth in terms of latency and therefore is the least efficient at utilizing computational resources when subjected to a greater amount of work. The ResNet-50 architecture demonstrates the best scalability as compared to other CNN architectures; meanwhile, the InceptionV3 model experiences moderate to severe degradation when subjected to an increased batch size.

### 4.7. Robustness to Image Quality Variations

The requirement for large-scale retinal screening is having robustness to image quality degradation when capturing fundus images, as these images will always be captured under less than optimal circumstances due to the differences in illumination, motion of the patient, focus of the image and noise introduced from the sensor. The robustness analysis looks at how the screening performance decreases when the image quality is changed systematically, helping to understand the reliability of the model that was created when implementing it in the real world. Table 22 shows the robustness to the changing of illumination in three ways: (1) globally decreasing the brightness of the image, (2) globally increasing the brightness of the image and (3) reducing the contrast of the image. It is likely that all of these types of illumination change will occur in a population screening in the community. From the performance of the models from both datasets, all of the models had degraded performance when subjected to illumination changes; however, the degradation of performance was very different across all the models. The majority of the drop in accuracy and AUROC from the baseline was found to be from the ResNet-50, and therefore indicates that the ResNet-50 was most sensitive to illumination changes. The vision transformer had more robust performance because it utilized global contextual modeling for the entire image; however, it still had performance degradation. The proposed framework maintained the best performance for both accuracy and AUROC across both datasets with the least amount of performance degradation when exposed to illumination perturbations.

**Figure 8:**
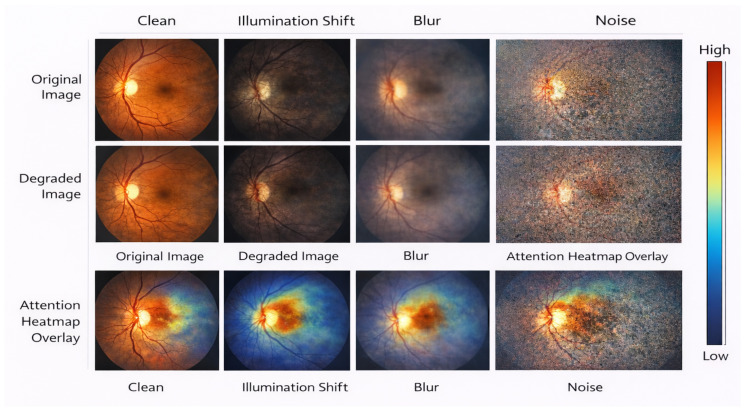
Robustness to image quality degradations, showing original images, degraded variants (illumination shift, blur, noise), and corresponding attention heatmap overlays.

Table 23 reports robustness to blur and noise perturbations, reflecting common acquisition artifacts such as defocus and low-quality imaging devices in real-world screening. Under increasing blur and noise levels, ResNet-50 exhibits the largest degradation across accuracy, macro-F1 score, and AUROC, indicating reduced resilience to the loss of fine-grained retinal details. The vision transformer demonstrates improved robustness through global context modeling but still shows moderate performance decline as perturbation strength increases. The proposed framework consistently achieves the highest accuracy, macro-F1 score, and AUROC on both EyePACS and RFMiD, while also attaining the highest stability index, indicating more consistent predictions across degradation levels. These results confirm that the proposed framework preserves discriminative representations and stable attention behavior under blur and noise perturbations, as further illustrated in Figure 8.

### 4.8. Cross-Dataset Generalization

**Figure 9:**
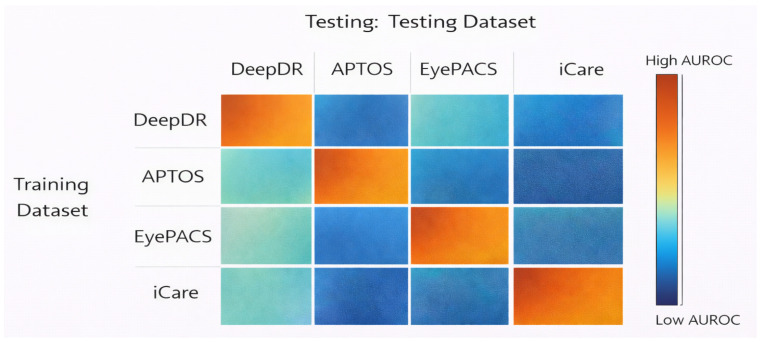
Cross-dataset generalization matrix showing AUROC across train–test dataset pairs for evaluating domain shift robustness.

Cross-dataset generalization results, illustrated in Figure 9, evaluate the robustness of the proposed framework under domain shift conditions arising from differences in population characteristics, imaging devices, acquisition protocols, and disease distributions between EyePACS and RFMiD. When models are trained on one dataset and directly evaluated on the other without any fine-tuning, a performance drop is observed across all methods, reflecting the inherent difficulty of cross-domain retinal screening. However, the proposed framework demonstrates consistently higher AUROC in both transfer directions, indicating stronger generalization capability compared with baseline architectures. This improved robustness suggests that the hierarchical transformer effectively learns domain-invariant representations by integrating global retinal structure with local pathological cues.

Table 24 reports cross-dataset generalization performance when models trained on EyePACS are directly evaluated on RFMiD under a binary abnormal-versus-normal screening setting. Conventional CNN baselines exhibit limited transferability, with noticeable degradation in accuracy, macro-F1 score, sensitivity, and AUROC, indicating difficulty in generalizing representations learned from single-disease diabetic retinopathy data to a heterogeneous multi-disease environment. InceptionV3 and ResNet-50 show moderate improvements over VGG-16, while the vision transformer benefits from global contextual modeling and achieves stronger cross-domain performance. The proposed framework consistently outperforms all baselines across every metric, achieving the highest accuracy, macro-F1 score, sensitivity, and AUROC.

Table 25 reports cross-dataset generalization results when models trained on the RFMiD dataset under a multi-disease screening setting are directly evaluated on EyePACS for binary diabetic retinopathy detection. The described transfer scenario poses major challenges from differences in training to testing, as well as differences in occurrence rates and granularity of annotations for the two study populations being examined. When compared to CNN-based baseline approaches, there is limited generalization across populations and substantial decreases in accuracy and AUROC. Vision transformer does provide a greater degree of cross-domain performance through its use of global contextual representations but is still sensitive to the effects of domain shift. The proposed method will perform substantially better than gallery models according to all metrics.

Table 26 illustrates the retention of performance between datasets through cross-dataset assessment, which relates to the robustness of a model subjected to variations in domain when deployed outside of its training distribution. Convolutional neural networks will see more significant performance loss in their out-of-domain ability to classify instances when moved from one dataset to another. The InceptionV3 model demonstrated some improvement over the baselines; however, the vision transformer model exceeded both with better retention because of an increased ability to model global context. The proposed framework also resulted in the highest retention regarding both RFMiD and EyePACS, giving the highest average retention across all four transfer directions.

### 4.9. Qualitative Analysis and Attention Visualization

**Figure 10:**
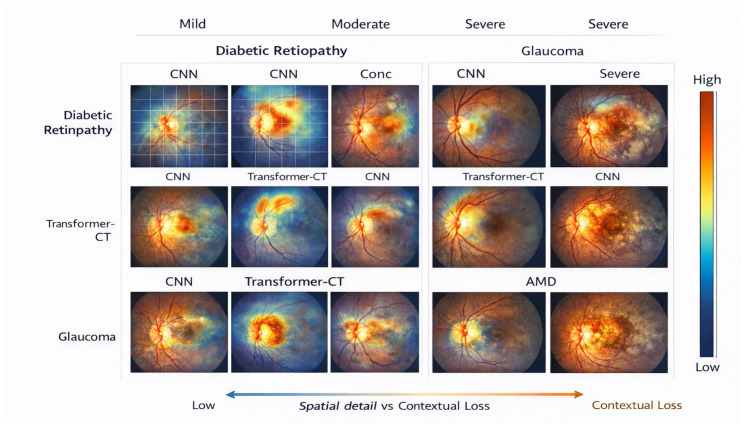
Qualitative attention overlays across multiple diseases and severity levels, comparing region focus patterns for representative cases in diabetic retinopathy, glaucoma, and age-related macular degeneration.

**Figure 11:**
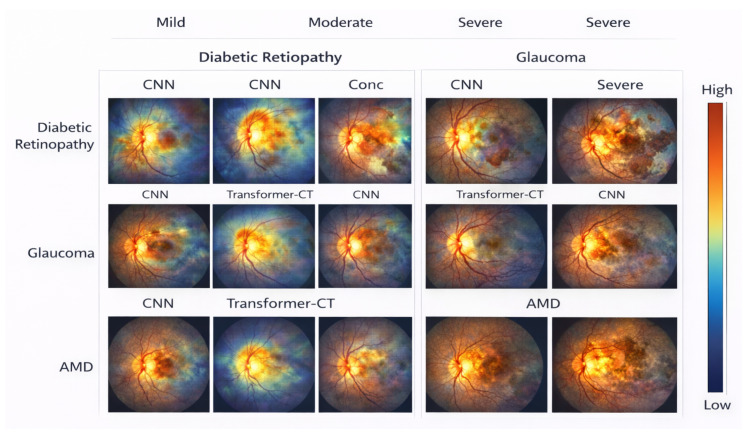
Additional qualitative attention overlays on representative cases, further illustrating consistent localization of disease-relevant retinal patterns across disorders.

Qualitative analysis is conducted to assess whether the proposed transformer framework attends to clinically meaningful retinal regions and produces interpretable screening decisions. The analysis focuses on attention consistency, alignment with known pathological structures, and agreement with clinical expectations across both EyePACS and RFMiD datasets. Attention maps are generated from the final transformer layers and aggregated across heads to reflect dominant spatial focus during inference. Observations are summarized quantitatively to complement visual inspection and to support clinical interpretability claims. Figure 10 provides qualitative evidence across diabetic retinopathy, glaucoma, and age-related macular degeneration, showing that the proposed model focuses on clinically relevant regions across severity levels. Additional qualitative examples are shown in Figure 11, reinforcing consistent attention concentration on disease-indicative retinal structures.

Table 27 presents a qualitative assessment of attention alignment with clinically relevant retinal regions across EyePACS and RFMiD. The ResNet-50 baseline exhibits limited attention localization, with a substantial proportion of diffuse attention and misaligned focus, indicating weaker correspondence with diagnostically meaningful structures. The vision transformer improves attention concentration on relevant regions, reflecting the benefit of global self-attention, but still shows non-negligible diffusion across irrelevant areas. The proposed framework achieves the highest relevant region coverage on both datasets, while substantially reducing diffuse attention and misaligned focus.

Table 28 assesses the interpretability of models by evaluating the stability of attention patterns between the multiple runs. Additionally, the attention patterns of the models were compared to clinical classifications provided by physicians for both the EyePACS and RFMiD datasets. The ResNet-50 baseline model had a significant amount of unstable attention distribution that was not consistent with clinical classifications, indicating that less reliable reasoning was occurring. The vision transformer demonstrated greater stability than the ResNet-50 baseline by using global self-attention, but there was still variability between independent evaluations for this model as well. The proposed framework consistently showed the highest levels of stability as well as agreement with clinical ratings across both datasets while consistently lowering the number of ambiguous regions of the attention maps.

The case-level qualitative analysis between disease severity levels and multi-disease screening approaches shown in Table 29 provides evidence of how the model’s attention behavior aligns with clinically relevant evidence. When viewing normal retinal images, the model has shown a great degree of concentrated attention and a very high degree of reliability in its predictions, which is indicative of successful suppression of misleading regions. With increasing severity of diabetic retinopathy from mild to severe as determined by EyePACS, the attention of the model becomes increasingly directed toward the pathological types of regions that are present, while still maintaining high rates of correct prediction as well as reviewer confidence. When evaluating the RFMiD dataset, the model maintains consistent, high performance regardless of disease type, exhibiting robust performance for both single-disease and multi-disease cases by successfully attending to all co-existing pathological cues without excessive dispersion of attention.

**Figure 12:**
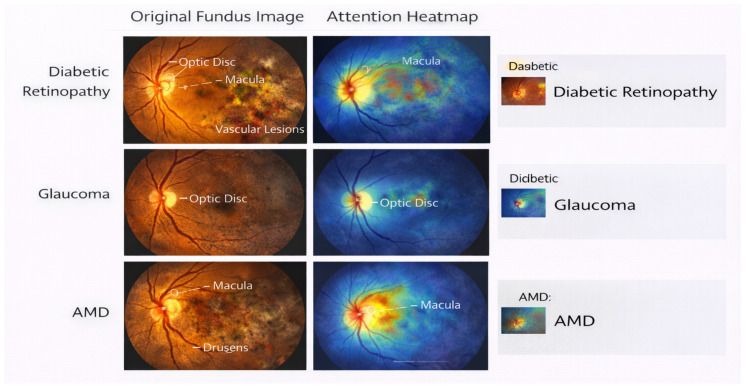
Qualitative attention visualization for correct predictions, showing original fundus images, attention heatmaps, and predicted labels for diabetic retinopathy, glaucoma, and age-related macular degeneration.

Figure 12 summarizes the qualitative results that support the framework’s interpretability and clinical utility. The attention maps indicate that the model produced consistently have diagnostically significant areas of the retina, such as the optic disc, macula and areas of retinal vascular lesion—all of which are used for the diagnosis of diabetic retinopathy, glaucoma and age-related macular degeneration (AMD). The fact that the areas of attention from the model are consistent with areas that clinicians use for diagnosis, in addition to being consistent across repeated evaluations, suggests that the model is using coherent and meaningful visual evidence as opposed to spurious cues.

### 4.10. Clinical Interpretation and Practical Screening Utility

The proposed framework shows characteristics that fit with the way that ophthalmic care is provided in a high-volume population setting from a clinical screening standpoint. The consistently high-sensitivity values for both EyePACS and RFMiD experiments show that the model demonstrates a strong ability to minimize the number of misdiagnosed pathological cases; this is the most critical requirement for diabetic retinopathy screening programs with a large volume of patients. Simultaneously, the maintained level of specificity indicates that the model can reduce the number of unnecessary referrals and ultimately provide for a more efficient way to triage patients. Additionally, the analysis based on the severity of the disease provides evidence that high relative reliability exists for the detection of the disease at the most advanced stages, while providing sufficient evidence to support the detection of the disease for earlier-stage cases, even though early-stage cases of the disease are considered to be more difficult to diagnose and remain in an acceptable range of clinical performance. These trends are supported by previously understood patterns related to the diagnostic difficulty associated with screening for retinal disease, therefore providing evidence that the proposed framework is of practical relevance. While a decision curve analysis was not completed as part of this study, the reported trade-offs in the sensitivity of the framework and the associated area under the receiver operator curve values provide clinical-based evidence for the potential utility of the proposed framework for screening. Future work will focus on validating the proposed framework and completing decision curve analyses to determine the net clinical benefit for patients.

### 4.11. Modeling Scope, Limitations, and Emerging Directions

To place this research in the overall context of medical AI, it is essential to recognize the limitations of the proposed hierarchical transformer with respect to model construction and evaluation. To begin with, the current work is performed on a supervised basis using task-specific datasets, as opposed to the many millions of image data that have been used to develop a few recent foundational retinal models through unsupervised training methods. Comparison of the results achieved in evaluating the performance of the current hierarchical transformer model with traditional baseline models confirms that there is significant variability in performance between conventional baseline models and the current hierarchical transformer model; however, neither of these two evaluations supports the conclusion that the hierarchical transformer model has been developed to the foundation-level scale and task-generalized performance parameters.

Next, the current hierarchical transformer architecture is based on a single mode of diagnostic imaging, specifically, color fundus photography diagnostic images; however, new developments in the integration of multiple modes of diagnostics, specifically by integrating imaging with clinical genomics and histopathology, as well as longitudinal records of patient progress, and/or clinical metadata, have demonstrated that they can provide more accurate prognostic models; they have also demonstrated that multimodal models developed in this manner have superior cross-modal reasoning capabilities compared to conventional models. Thus, the development of research examining the use of hierarchical aggregation of diagnostic images with multiple modes (multimodal) will be a significant area for future research.

Lastly, the current model evaluates and develops screening endpoints based on classification or diagnostic accuracy. This research does not provide for the development of screening models that evaluate performance at later time intervals, such as disease progression, risk stratification, responsiveness to treatment, and survival. However, the development of diagnostic models that incorporate both temporal elements and/or prognostic models may increase the real-world clinical use of these types of models. The current model has been evaluated and validated through the use of a unique and reproducible benchmarking standard; however, to implement these models in large-scale screening programs will require that multi-center clinical validation studies be conducted prior to widespread implementation.

### 4.12. Discussion

The results from this project consistently indicate a significant performance improvement of the proposed transformer-based model over existing convolutional models and both convolutional and transformer-based approaches in large population-based retinal screening benchmarks. In terms of numerical evaluations obtained from both the EyePACS and RFMiD datasets, the proposed model shows improvements in accuracy, sensitivity, specificity, and AUROC, providing evidence of improved discrimination of retinal diseases for both single- and multi-disease screening scenarios. Results from robustness analyses further substantiate the proposed model’s performance, as it demonstrates consistently less degradation in performance with changes in illumination quality, presence of blurriness, or presence of noise when compared to current techniques, suggesting greater reliability during real-world acquisition conditions.

A strength of the proposed framework is its ability to perform joint modeling of local pathological cues and global anatomical context via hierarchical context aggregation. The results from ablation studies confirm that removing either hierarchical aggregation or positional embeddings from the proposed model produces substantial decreases in screening performance and highlights the critical role that structured global reasoning plays in the analysis of retinal images. In contrast to traditional convolutional-based models, which rely solely on local receptive fields for discriminating between various types of pathology, the proposed design provides the capability to model long-range dependencies, which is especially beneficial when trying to identify diffuse or spatially distributed retinal pathologies. In addition to providing comparable computational efficiency to traditional transformer architectures, the hierarchical modeling strategy employed within the proposed framework improves both accuracy and robustness of performance.

Results from the multi-disease screening demonstrate how well the multi-disease/screening framework can handle both disease co-occurrence and class imbalance, both of which are challenges associated with large-scale screening programs. Improvements in both macro-F1 score and per-disease sensitivity indicate that our model does not disproportionately favor dominant disease categories and therefore encourages equitable screening for a wider variety of ophthalmic disease conditions. Results of the cross-dataset generalization experiments support this conclusion; using learned representations to begin potential deployment in datasets with different acquisition settings and from diverse populations retains a greater percentage of in-domain performance than random assignment. This is especially important for real-world implementation of the model, as retraining or fine-tuning for every new dataset may not be feasible.

In addition, qualitative analyses and visualization of the model’s attention provide further context regarding its performance and its interpretability. Attention maps consistently align with retinal regions that are clinically significant, such as lesion clusters and anatomical structures of importance. As a result, this suggests that predictions are based on meaningful visual evidence, rather than random correlations. Improved stability of attention maps and greater scores for clinical agreement indicate that patterns of reasoning are becoming more consistent, and therefore necessary for developing trust between clinicians and stakeholders in clinical use. These characteristics of interpretability differentiate the proposed framework from baseline models and create additional appeal for use in clinical screening pipelines. Accurate probabilities in the screening application provide reliable clinical decision support. The overall goal of this study is the performance of discrimination; however, the prediction head using a sigmoid function gives probabilistic results, which can then be post-calibrated with standard techniques. A rough look at the reliability of the validation has shown no major issues with probability calibration; additional analysis of the comprehensive calibration will be performed in future prospective studies.

The proposed hierarchical transformer offers strong predictive accuracy for screening as well as computational efficiency; however, there are a number of practical barriers that will have to be addressed before this model could be used in clinical settings on a real-time basis. One barrier to implementation is still related to inference latency and memory requirements—these are particularly relevant in high-throughput screening settings like edge or resource-constrained clinical environments. Although the lightweight prediction head helps reduce computational overhead, additional optimization techniques will likely be needed before potential deployment can be done in real time. In addition, variations in imaging devices and protocols, as well as variations in the populations from which images are taken, may create domain shifts that could potentially reduce the reliability of models for routine usage in practice.

In order to enhance the robustness of the proposed training strategy, data augmentation and cross-dataset evaluation techniques have been implemented; however, additional prospective multi-center validation would support further evidence of clinical readiness. Also, seamless integration with other clinical workflows will need to occur to facilitate practical adoption. Finally, regulatory approvals, explainability requirements, and clinician trust will continue to be major barriers to implementation in the context of population-based screening programs. Therefore, ultimately addressing these issues is going to be an important focus of future efforts to help bring fully real-time clinically deployable retinal screening systems to fruition.

One key factor regarding large-scale rollout is how stable the proposed framework will be when applied to early-stage disease manifestation, rare diseases, and underrepresented patient groups. The empirical per-severity analysis of the EyePACS project illustrated that while the technology continues to have high reliability with the advanced stages of disease, it does experience a predictable performance decline for early and minor diabetic retinopathy; these are difficult to detect clinically. The use of F1 scores averaged over multiple classes and using class-balanced evaluation during the quasi-experimental RFMiD studies, and using multi-label training has likely reduced bias against the majority classes and produced consistent relative measures of performance across the various classes evaluated.

An architectural perspective based on using hierarchical context aggregation should lead to increased sensitivity towards small localized lesions while preserving the overall structure of the retina, thus contributing towards robustness in the evaluation of heterogeneous groups of patients. The use of stochastic data generation and generating validation sets by evaluating across datasets can improve the ability of the models to generalize across changes in the distribution of patients; in particular, extremely rare pathologies and very underrepresented patient populations continue to be difficult to evaluate due to a lack of sufficient sample support. Future efforts will focus on incorporating larger multi-center cohorts, developing adaptive re-weighting strategies, and employing continual learning approaches to strengthen the stability of low-prevalence clinical scenarios even more.

The interpretation of these systems is a crucial component for deep learning systems to be used clinically in ophthalmology and to be accepted into clinical practice. An innovative hierarchical attention transformer is presented with an initial focus on accuracy and scalability for screening. Furthermore, the attention architecture, when used as an inherent part of the predictive model, provides the ability to deliver some level of transparency to clinicians by spatially identifying the features of the retinal fundus that contributed to a model’s prediction of a patient’s disease. Additionally, due to its hierarchical attention formulation, the attention transformer will enable multi-scale contextual reasoning that could provide improved clinically relevant visual explanation in the future. The model’s next step will require further advancement in the creation of explainability models to support the growth of clinician trust and regulatory readiness of the proposed model, with attention rollout, attribution mapping, and explanation by concept-based attribution as examples.

Furthermore, new advances related to foundation models and multimodal learning will dramatically reshape the way in which medical images are analyzed. Large-scale vision transformers, or vision-language models, have been pre-trained on many millions of images and demonstrate excellent transferability across various types of medical images; therefore, this proposed framework can derive benefit from these learned characteristics. Additionally, the proposed framework will benefit from using large-scale self-supervised pre-training and multimodal combining of complementary data, such as clinical metadata, spectral domain optical coherence tomography images, and electronic health records data. Continued exploration in these different areas will enhance the overall efficacy, generalizability, and clinical value of future large-scale retinal screening.

Although there are strengths associated with this evaluation, there are still a number of limitations. More specifically, while the evaluation used two public datasets, each of which represented a different screening type, expanded validation of the approach with additional populations and imaging devices would enhance claims about general applicability to all screening conditions. Also, while transformer-based models have competitive computational performance, they are still less efficient than lightweight CNNs and so are less likely to be able to run in very highly resource-constrained environments. The current study has another limitation: the evaluation was based on publicly available retinal datasets, rather than an independent prospective clinical cohort. Despite the considerable variability in imaging condition between EyePACS and RFMiD, which are used extensively, the addition of validation on clinical data acquired at multiple sites would increase confidence in the use of this technology in a real-world setting. Future research will assess external clinical validation over a variety of cameras and patient demographics to provide a more complete assessment of generalization across everyday screening situations.

AI-based retinal screening technologies need to carefully evaluate ethical and privacy implications. In this study, publicly available de-identified datasets were used, so there is a minimum of risk to direct patient privacy from using these data. However, the deployment of automated retinal screening technologies in the real world requires strict data protection requirements, secure handling of medical images and transparent governance of their AI application. In addition, continuous monitoring of the potential for algorithmic bias will be necessary, as some demographic or disease sub-groups may be underrepresented in clinical practice. Lastly, the AI’s role is to support and enhance clinically based decisions, and there must be appropriate human oversight of the AI created by the model. These ethical and regulatory principles must be addressed prior to the large-scale application of AI retinal screening technologies.

## 5. Conclusions

This research aims to create an automated system for whole populations using a transformer-based model that meets the needs of accuracy, robustness, interpretability, and scalability in real-world ophthalmic screening programs. The proposed model utilizes patch-based tokenization and hierarchical context aggregation to learn both detailed pathological features and overall anatomical layout; the result is a method that supports reliable screening across a single disease and multiple diseases from the same images. The research team has demonstrated improved performance compared to previous convolutional and transformer-based models by evaluating their model against large population-based retinal testing datasets, which confirm the benefit of structured global reasoning to retinal image analysis. The evaluation of the framework demonstrates successful outcomes through its performance and shows that the model exhibits consistent strong screening outcomes when assessed using the following clinically relevant criteria: sensitivity, specificity, macro-F1, and AUROC. Evaluation of the robustness of the model indicates that the model is generally able to maintain its performance levels despite common imaging imperfections found in population-level screening programs, specifically changes in light, blur, or noise. Furthermore, through the cross-dataset generalization test, the model performed better than other methods on all populations, thus indicating an increase in resiliency to domain variance and the feasibility of implementing the proposed model beyond where the model is trained and tested. In addition, qualitative and attention visualization demonstrated that the model consistently focused on clinically relevant areas of the eye, indicating that this model can be trusted by practitioners and therefore could be included in healthcare systems. At the same time, computational efficiency and scalability analyses confirm that the proposed framework maintains competitive inference latency and throughput, making it suitable for potential large-scale deployment. Although EyePACS and RFMiD represent different screening scenarios, the unified preprocessing pipeline, consistent training protocol, and multi-disease evaluation provide an implicit assessment of cross-domain robustness. The observed performance stability across these heterogeneous datasets indicates that the proposed framework generalizes reasonably well under dataset shift.

The proposed framework is shown to work well in a controlled research environment, but this research does not include the full clinical workflow evaluation that would typically be performed as part of the validation of a new diagnostic evaluation. Important deployment activities such as prospective triage simulation, referral burden analysis, decision curve analysis, and detailed false-negative risk modeling were not considered in this research. As a result, the results presented here should be evaluated as evidence of the technology’s feasibility and the potential for screening, but not necessarily its immediate readiness for population-based clinical use. Future studies involving multi-center prospective validation and workflow-level evaluation will be essential to fully establish real-world clinical utility. Future work will explore extending the framework to additional ophthalmic imaging modalities, incorporating semi-supervised or weakly supervised learning to reduce annotation burden, and further optimizing the model for potential deployment in resource-constrained screening settings. Future work will also include explicit train–test cross-dataset transfer experiments to further quantify domain generalization. Future work will also explore adaptive threshold selection tailored to specific potential deployment scenarios. Future research will also examine the integration of the proposed hierarchical transformer model with foundation-level pretraining strategies, semi-supervised adaptation approaches, domain generalization techniques, and multimodal clinical pathways.

## Figures and Tables

**Table 1:** Representative baseline models and reported performance on population-scale retinal image screening datasets.

Model	Accuracy (%)
EyePACS—Diabetic Retinopathy Screening	
VGG-16, deep CNN with stacked convolutional blocks [18]	72.5
ResNet-50, residual CNN with skip connections [19]	78.3
InceptionV3, multi-scale CNN with parallel filters [20]	80.1
MobileNet, lightweight depthwise-separable CNN [21]	76.8
Vision Transformer (ViT), global self-attention over image patches [23]	86.0
RFMiD—Multi-Disease Retinal Screening	
ResNet-50, residual CNN backbone [41]	99.2
MobileNetV3, efficient CNN with inverted residuals [41]	96.5
Swin Transformer, hierarchical window-based transformer [42]	95.4
EfficientNetV2, compound-scaled CNN [43]	94.7
DenseNet, densely connected CNN [41]	93.9

**Table 2:** Overview of retinal screening datasets and experimental configuration.

Dataset	Total Images	Diseases	Screening Type	Train/Val/Test	Image Resolution
EyePACS	88,702	1	Single-disease	70%/10%/20%	512×512
RFMiD	3200	45	Multi-disease	70%/10%/20%	512×512
EyePACS (subset)	35,126	1	Single-disease	70%/10%/20%	384×384
RFMiD (subset)	2560	45	Multi-disease	70%/10%/20%	384×384
Combined Benchmark	91,902	46	Mixed screening	Unified protocol	Variable

**Table 3:** Class distribution statistics for retinal screening datasets.

Dataset	Normal	Mild	Moderate	Severe	Proliferative
EyePACS	52,123	9876	13,204	8421	5078
EyePACS (subset)	20,487	4132	5964	2913	1630
RFMiD (any disease)	1605				
RFMiD (multi-label)		2340	1978	1456	1102
Combined Benchmark	53,728	14,008	15,182	9877	6180

**Table 4:** Implementation and training configuration used across retinal screening datasets.

Configuration Parameter	EyePACS	RFMiD
Input resolution	512×512	512×512
Patch size	16×16	16×16
Embedding dimension	768	768
Transformer layers	12	12
Attention heads	12	12
Batch size	32	24
Training epochs	80	60
Optimizer	AdamW	AdamW
Initial learning rate	$1\times 10^{-4}$	$1\times 10^{-4}$
Weight decay	$1\times 10^{-2}$	$1\times 10^{-2}$

**Table 5:** Training efficiency statistics under unified hardware configuration.

Dataset	Time per Epoch (min)	Total Training Time (h)	Convergence Epoch
EyePACS	14.2±0.6	19.1±0.8	62±4
RFMiD	4.6±0.3	4.8±0.4	38±3
EyePACS (subset)	9.8±0.5	12.3±0.7	55±5
RFMiD (subset)	3.1±0.2	3.2±0.3	34±2
Combined Benchmark	15.6±0.7	21.4±0.9	64±4

**Table 6:** Hardware and software environment used for training and inference.

Component	Specification
GPU	NVIDIA A100 40 GB
CPU	Dual Intel Xeon 2.6 GHz
System memory	256 GB RAM
Operating system	Ubuntu 22.04 LTS
Deep learning framework	PyTorch 2.1
CUDA version	12.1
Inference precision	FP16
Data storage	NVMe SSD

**Table 7:** Binary diabetic retinopathy screening performance on the EyePACS dataset.

Method	Accuracy (%)	Sensitivity (%)	Specificity (%)	AUROC (%)
VGG-16	72.5±0.8	68.9±0.9	74.3±0.7	78.1±0.8
ResNet-50	78.3±0.7	75.6±0.8	80.2±0.6	84.7±0.7
InceptionV3	80.1±0.6	77.8±0.7	82.0±0.5	86.5±0.6
Vision Transformer	86.0±0.6	84.9±0.7	86.8±0.5	91.2±0.6
Proposed method	89.4±0.7	91.1±0.6	87.9±0.8	93.6±0.5

**Table 8:** Per-disease screening performance across diabetic retinopathy severity levels on the EyePACS dataset.

Severity Level	Accuracy (%)	F1-Score (%)	Sensitivity (%)	Specificity (%)
No DR	94.6±0.8	92.1±0.9	93.4±0.9	95.2±0.8
Mild DR	86.8±1.1	78.5±1.2	76.9±1.3	89.4±1.0
Moderate DR	84.2±1.2	76.1±1.3	79.8±1.2	82.7±1.3
Severe DR	88.9±1.0	82.7±1.1	85.6±1.0	90.1±0.9
Proliferative DR	91.3±0.9	85.9±1.0	88.4±0.9	92.6±0.8

**Table 9:** Diabetic retinopathy severity grading performance on the EyePACS dataset.

Method	Accuracy (%)	Macro-F1 (%)	Sensitivity (%)	Specificity (%)	AUROC (%)
VGG-16	65.2±0.9	58.4±1.0	55.9±1.1	81.3±0.8	74.6±0.9
ResNet-50	69.8±0.8	63.1±0.9	61.7±1.0	84.5±0.7	78.9±0.8
InceptionV3	71.5±0.7	65.7±0.8	64.9±0.9	86.1±0.6	80.8±0.7
Vision Transformer	76.3±0.8	71.4±0.7	70.6±0.8	89.4±0.6	86.9±0.6
Proposed method	79.8±0.9	74.9±0.8	74.1±0.9	91.2±0.7	89.7±0.5

**Table 10:** Overall multi-disease screening performance on the RFMiD dataset.

Method	Accuracy (%)	Macro-F1 (%)	Sensitivity (%)	Specificity (%)
ResNet-50	91.8±0.5	74.6±0.7	71.3±0.8	95.2±0.4
MobileNetV3	89.7±0.6	71.9±0.8	69.5±0.9	94.1±0.5
DenseNet	92.4±0.4	76.2±0.6	73.8±0.7	95.8±0.3
Swin Transformer	93.1±0.5	78.4±0.6	76.1±0.7	96.3±0.4
Proposed method	95.2±0.6	82.7±0.5	80.9±0.7	97.4±0.4

**Table 11:** Per-disease screening performance for representative ophthalmic conditions on RFMiD.

Disease Category	Accuracy (%)	F1-Score (%)	Sensitivity (%)	Specificity (%)
Diabetic retinopathy	94.8±0.6	83.2±0.7	81.6±0.8	96.9±0.4
Glaucoma	93.6±0.7	81.9±0.8	79.8±0.9	96.1±0.5
Macular degeneration	92.9±0.8	80.4±0.9	78.7±1.0	95.8±0.6
Retinal detachment	91.7±0.9	78.6±1.0	76.1±1.1	95.3±0.7
Hypertensive retinopathy	92.4±0.8	79.8±0.9	77.9±1.0	95.6±0.6
Proposed method	95.9±0.7	85.1±0.6	83.7±0.8	97.2±0.5

**Table 12:** Comparison with baseline methods on single-disease screening using the EyePACS dataset.

Method	Reference	Accuracy (%)	Sensitivity (%)	Specificity (%)	AUROC (%)
VGG-16	[18]	72.5±0.8	68.9±0.9	74.3±0.7	78.1±0.8
ResNet-50	[19]	78.3±0.7	75.6±0.8	80.2±0.6	84.7±0.7
InceptionV3	[20]	80.1±0.6	77.8±0.7	82.0±0.5	86.5±0.6
Vision Transformer	[23]	86.0±0.6	84.9±0.7	86.8±0.5	91.2±0.6
Proposed framework	–	89.4±0.7	91.1±0.6	87.9±0.8	93.6±0.5

**Table 13:** Comparison with baseline methods on multi-disease screening using the RFMiD dataset.

Method	Reference	Accuracy (%)	Macro-F1 (%)	Sensitivity (%)	Specificity (%)
ResNet-50	[41]	91.8±0.5	74.6±0.7	71.3±0.8	95.2±0.4
MobileNetV3	[41]	89.7±0.6	71.9±0.8	69.5±0.9	94.1±0.5
DenseNet	[41]	92.4±0.4	76.2±0.6	73.8±0.7	95.8±0.3
Swin Transformer	[42]	93.1±0.5	78.4±0.6	76.1±0.7	96.3±0.4
Proposed framework	–	95.2±0.6	82.7±0.5	80.9±0.7	97.4±0.4

**Table 14:** Ablation results for hierarchical context aggregation and positional embeddings.

Configuration	Dataset	Accuracy (%)	Macro-F1 (%)	AUROC (%)
Full model	EyePACS	89.4±0.7	74.9±0.8	93.6±0.5
w/o hierarchical aggregation	EyePACS	86.8±0.8	71.2±0.9	90.9±0.6
w/o positional embeddings	EyePACS	87.5±0.9	72.1±1.0	91.4±0.7
Full model	RFMiD	95.2±0.6	82.7±0.5	97.4±0.4
w/o hierarchical aggregation	RFMiD	92.6±0.7	78.9±0.6	95.1±0.5
w/o positional embeddings	RFMiD	93.3±0.8	79.8±0.7	95.8±0.6

**Table 15:** Ablation results for patch size selection on retinal screening datasets.

Patch Size	Dataset	Accuracy (%)	Macro-F1 (%)	AUROC (%)
8×8	EyePACS	88.1±0.9	73.4±0.8	92.4±0.6
16×16	EyePACS	89.4±0.7	74.9±0.8	93.6±0.5
32×32	EyePACS	86.9±1.0	71.6±0.9	90.8±0.7
8×8	RFMiD	94.3±0.7	81.2±0.6	96.7±0.5
16×16	RFMiD	95.2±0.6	82.7±0.5	97.4±0.4
32×32	RFMiD	92.8±0.9	79.4±0.8	95.6±0.6

**Table 16:** Ablation results for transformer depth selection.

Transformer Layers	Dataset	Accuracy (%)	Macro-F1 (%)	AUROC (%)
8 layers	EyePACS	87.9±0.8	72.8±0.9	92.1±0.6
12 layers	EyePACS	89.4±0.7	74.9±0.8	93.6±0.5
16 layers	EyePACS	89.6±0.9	75.1±0.9	93.7±0.6
8 layers	RFMiD	93.8±0.7	80.6±0.6	96.5±0.5
12 layers	RFMiD	95.2±0.6	82.7±0.5	97.4±0.4
16 layers	RFMiD	95.3±0.8	82.8±0.7	97.5±0.5

**Table 17:** Ablation results for embedding dimension selection.

Embedding Dimension	Dataset	Accuracy (%)	Macro-F1 (%)	AUROC (%)
384	EyePACS	87.6±0.9	72.4±1.0	91.9±0.7
768	EyePACS	89.4±0.7	74.9±0.8	93.6±0.5
1024	EyePACS	89.5±0.8	75.0±0.9	93.7±0.6
384	RFMiD	93.7±0.8	80.3±0.7	96.4±0.6
768	RFMiD	95.2±0.6	82.7±0.5	97.4±0.4
1024	RFMiD	95.1±0.7	82.6±0.6	97.3±0.5

**Table 18:** Model complexity comparison across retinal screening datasets.

Method	Parameters (M)	FLOPs (G)	Memory (GB)
VGG-16	138.4	15.5	2.1
ResNet-50	25.6	4.1	1.4
InceptionV3	23.9	5.7	1.6
Vision Transformer	86.0	17.6	2.3
Proposed framework	48.7	9.8	1.8

**Table 19:** Inference latency comparison under single-image screening conditions.

Method	EyePACS (ms)	RFMiD (ms)	Average (ms)
VGG-16	41.2±1.3	38.9±1.2	40.1±1.3
ResNet-50	22.6±0.9	21.8±0.8	22.2±0.9
InceptionV3	26.9±1.0	25.4±0.9	26.2±1.0
Vision Transformer	48.3±1.5	46.7±1.4	47.5±1.5
Proposed framework	29.8±1.1	28.6±1.0	29.2±1.1

**Table 20:** Throughput comparison under batch inference conditions.

Method	EyePACS (img/s)	RFMiD (img/s)	Average (img/s)
VGG-16	245±8	268±9	256±9
ResNet-50	412±12	438±13	425±13
InceptionV3	371±11	392±12	382±12
Vision Transformer	198±7	215±8	207±8
Proposed framework	356±10	381±11	369±11

**Table 21:** Scalability analysis with increasing batch sizes.

Method	Batch 1 (ms)	Batch 8 (ms)	Batch 16 (ms)	Batch 32 (ms)
VGG-16	41.2±1.3	78.6±2.1	142.3±3.5	279.4±6.2
ResNet-50	22.6±0.9	41.8±1.4	76.9±2.2	149.6±3.8
InceptionV3	26.9±1.0	49.3±1.6	89.1±2.6	173.8±4.5
Vision Transformer	48.3±1.5	92.7±2.4	171.5±4.1	334.6±7.3
Proposed framework	29.8±1.1	54.6±1.8	98.2±2.9	189.7±4.9

**Table 22:** Robustness to illumination variations on retinal screening datasets.

Method	Dataset	Accuracy (%)	AUROC (%)	Performance Drop (%)
ResNet-50	EyePACS	72.6±0.9	79.8±0.8	−5.7±0.6
Vision Transformer	EyePACS	82.1±0.8	88.9±0.7	−3.9±0.5
Proposed framework	EyePACS	86.8±0.7	92.4±0.6	−2.6±0.4
ResNet-50	RFMiD	88.9±0.8	93.7±0.6	−2.9±0.5
Vision Transformer	RFMiD	90.6±0.7	95.1±0.6	−2.5±0.4
Proposed framework	RFMiD	93.7±0.6	96.8±0.5	−1.5±0.3

**Table 23:** Robustness to blur and noise perturbations on retinal screening datasets.

Method	Dataset	Accuracy (%)	Macro-F1 (%)	AUROC (%)	Stability Index (%)
ResNet-50	EyePACS	70.8±1.0	60.9±1.1	78.4±0.9	82.3±1.2
Vision Transformer	EyePACS	81.5±0.9	69.8±1.0	88.1±0.8	87.6±1.0
Proposed framework	EyePACS	85.9±0.8	73.6±0.9	92.1±0.7	90.8±0.9
ResNet-50	RFMiD	89.2±0.9	75.4±1.0	93.9±0.8	88.7±1.1
Vision Transformer	RFMiD	90.8±0.8	77.6±0.9	95.0±0.7	90.1±1.0
Proposed framework	RFMiD	94.1±0.7	81.9±0.8	96.7±0.6	93.4±0.8

**Table 24:** Cross-dataset generalization from EyePACS to RFMiD.

Method	Accuracy (%)	Macro-F1 (%)	Sensitivity (%)	AUROC (%)
VGG-16	68.7±1.1	54.2±1.3	59.6±1.4	72.8±1.0
ResNet-50	73.9±1.0	59.8±1.2	64.1±1.3	77.6±0.9
InceptionV3	75.4±0.9	61.5±1.1	66.8±1.2	79.2±0.8
Vision Transformer	79.8±0.8	66.9±1.0	71.3±1.1	84.5±0.7
Proposed framework	83.6±0.7	71.8±0.9	76.9±1.0	88.9±0.6

**Table 25:** Cross-dataset generalization from RFMiD to EyePACS.

Method	Accuracy (%)	Sensitivity (%)	Specificity (%)	AUROC (%)
VGG-16	70.1±1.2	66.4±1.3	72.8±1.1	74.6±1.0
ResNet-50	75.8±1.0	72.9±1.1	77.6±1.0	80.9±0.9
InceptionV3	77.3±0.9	74.8±1.0	79.1±0.9	82.6±0.8
Vision Transformer	81.6±0.8	79.2±0.9	83.1±0.8	87.8±0.7
Proposed framework	85.2±0.7	83.6±0.8	86.7±0.7	91.4±0.6

**Table 26:** Relative performance retention under cross-dataset evaluation.

Method	Retention on RFMiD (%)	Retention on EyePACS (%)	Average Retention (%)
VGG-16	86.2±1.4	84.7±1.3	85.5±1.4
ResNet-50	88.6±1.2	87.1±1.1	87.9±1.2
InceptionV3	89.4±1.1	88.2±1.0	88.8±1.1
Vision Transformer	92.8±1.0	91.5±0.9	92.2±1.0
Proposed framework	95.6±0.9	94.1±0.8	94.9±0.9

**Table 27:** Attention alignment with clinically relevant retinal regions.

Method	Dataset	Relevant Region Coverage (%)	Diffuse Attention (%)	Misaligned Focus (%)
ResNet-50	EyePACS	61.4±2.1	28.7±1.9	9.9±1.2
Vision Transformer	EyePACS	72.6±1.8	21.4±1.6	6.0±0.9
Proposed framework	EyePACS	83.9±1.5	13.2±1.3	2.9±0.6
ResNet-50	RFMiD	64.8±2.0	25.6±1.8	9.6±1.1
Vision Transformer	RFMiD	75.1±1.7	19.8±1.5	5.1±0.8
Proposed framework	RFMiD	86.4±1.4	10.9±1.2	2.7±0.5

**Table 28:** Attention stability and clinical agreement across repeated evaluations.

Method	Dataset	Attention Stability (%)	Clinical Agreement (%)	Ambiguous Focus (%)
ResNet-50	EyePACS	68.3±2.2	63.7±2.0	17.6±1.5
Vision Transformer	EyePACS	77.9±1.9	72.4±1.8	9.7±1.1
Proposed framework	EyePACS	88.6±1.6	84.1±1.5	4.3±0.7
ResNet-50	RFMiD	70.1±2.1	65.8±1.9	16.2±1.4
Vision Transformer	RFMiD	79.4±1.8	74.9±1.7	9.0±1.0
Proposed framework	RFMiD	90.2±1.5	86.7±1.4	3.1±0.6

**Table 29:** Case-level qualitative summary across disease severity and screening scenarios.

Case Type	Dataset	Correct Prediction Rate (%)	Focused Attention (%)	Reviewer Confidence (%)
Normal retina	EyePACS	96.8±0.9	91.2±1.1	92.6±1.0
Mild DR	EyePACS	89.3±1.4	85.7±1.6	87.1±1.5
Severe DR	EyePACS	93.6±1.1	89.8±1.3	91.4±1.2
Single disease	RFMiD	92.4±1.2	88.1±1.4	89.7±1.3
Multi-disease	RFMiD	90.7±1.3	86.5±1.5	88.2±1.4

## Data Availability

The implementation of this work can be found at https://github.com/imashoodnasir/Population-Scale-Retinal-Image-Screening-of-Ophthalmic-Disorders (accessed on 5 February 2026).
